# Mesenchymal stem cells from perinatal tissues promote diabetic wound healing via PI3K/AKT activation

**DOI:** 10.1186/s13287-025-04141-8

**Published:** 2025-02-08

**Authors:** Jiawei Huang, Qingwen Deng, Lai Ling Tsang, Guozhu Chang, Jinghui Guo, Ye Chun Ruan, Chi Chiu Wang, Gang Li, Hon Fai Chan, Xiaohu Zhang, Xiaohua Jiang

**Affiliations:** 1 School of Biomedical Sciences, Faculty of Medicine; CUHK-GIBH CAS Joint Research Laboratory On Stem Cell and Regenerative Medicine; Key Laboratory for Regenerative Medicine of the Ministry of Education of China, The Chinese University of Hong Kong, Hong Kong SAR, China; 2 Sichuan University-The Chinese University of Hong Kong Joint Laboratory for Reproductive Medicine, West China Second University Hospital, Sichuan University, Chengdu, 610041 Sichuan China; 3 The Chinese University of Hong Kong, Shenzhen Research Institute, Shenzhen, 518000 China; 4 Institute for Tissue Engineering and Regenerative Medicine, The Chinese University of Hong Kong, Hong Kong SAR, China; 5 School of Medicine, The Chinese University of Hong Kong, Shenzhen, 518172 Guangdong China; 6 Department of Biomedical Engineering, The Hong Kong Polytechnic University, Hong Kong SAR, China; 7 Department of Obstetrics and Gynaecology, Faculty of Medicine, The Chinese University of Hong Kong; Reproduction and Development, Li Ka Shing Institute of Health Sciences, The Chinese University of Hong Kong, Hong Kong SAR, China; 8 Department of Orthopaedics & Traumatology, Faculty of Medicine, The Chinese University of Hong Kong, Prince of Wales Hospital, Shatin, Hong Kong SAR, China

**Keywords:** Diabetic wound healing, Placenta, MSCs, Hydrogel, PI3K/AKT

## Abstract

**Background:**

Diabetic foot ulcers (DFUs) represent a major complication of diabetes, often leading to poor healing outcomes with conventional treatments. Mesenchymal stem cell (MSC) therapies have emerged as a promising alternative, given their potential to modulate various pathways involved in wound healing. This study evaluates and compares the therapeutic potential of MSCs derived from perinatal tissues—human umbilical cord MSCs (hUCMSCs), human chorionic villi MSCs (hCVMSCs), and human decidua basalis MSCs (hDCMSCs)—in a diabetic wound healing model.

**Methods:**

We performed in vitro and in vivo studies to compare the efficacy of hUCMSCs, hCVMSCs, and hDCMSCs. Mass spectrometry was used to analyze the secreted proteins of the MSCs. We incorporated the MSCs into a polyethylene glycol diacrylate (PEGDA) and sodium alginate (SA) hydrogel matrix with collagen I (Col-I) to evaluate their effects on wound healing.

**Results:**

All three types of MSCs promoted wound healing, with hUCMSCs and hCVMSCs showing stronger effects compared to hDCMSCs. Both hUCMSCs and hCVMSCs demonstrated robust wound healing kinetics, with enhanced keratinocyte proliferation (KRT14^+^/Ki67^+^ cells), maturation (KRT10/KRT14 ratio), and angiogenesis. In vitro studies demonstrated that the MSC-derived secretome enhanced keratinocyte proliferation and migration, endothelial cell function and stem cell recruitment, indicating robust paracrine effects. Mass spectrometry revealed a conserved set of proteins including THBS1 (thrombospondin 1), SERPINE1 (serpin family E member 1), ANXA1 (annexin A1), LOX (lysyl oxidase), and ITGB1 (integrin beta-1) which are involved in extracellular matrix (ECM) organization and wound healing, with the PI3K/AKT signaling pathway playing a central role. The PEGDA/SA/Col-I hydrogel demonstrated a unique balance of mechanical and biological properties and an optimal environment for MSC viability and function. Application of either hUCMSC- or hCVMSC-laden hydrogels resulted in accelerated wound closure, improved re-epithelialization, increased collagen deposition, and enhanced vascularization in vivo.

**Conclusions:**

MSCs From perinatal tissues particularly hUCMSCs and hCVMSCs significantly enhance diabetic wound healing through PI3K/AKT pathway activation while hDCMSCs exhibited weaker efficacy. The PEGDA/SA/Col-I hydrogel supports MSC viability and function offering a promising scaffold for DFU treatment. These findings underscore the potential of specific perinatal MSCs and optimized hydrogel formulations in advancing diabetic wound care.

**Supplementary Information:**

The online version contains supplementary material available at 10.1186/s13287-025-04141-8.

## Introduction

Diabetic foot ulcers (DFUs) are common and serious complications that arise from uncontrolled diabetes. As reported by the International Diabetes Federation, approximately 19–34% of the diabetic patients worldwide will experience DFU at some point in their lives [1]. DFUs pose significant health risks, with 10% of affected individuals succumbing to mortality within the first year of diagnosis and 20–30% requiring lower limb amputations, ranging from minor (below the ankle) to major (above the ankle) or even multiple amputations [2]. The pathogenesis of DFUs is characterized by a classic triad comprising neuropathy, ischemia, and infection, each contributing to the development and progression of the condition. Neuropathy results in sensory loss and autonomic dysfunction, leading to reduced pain perception and impaired protective sensations. Ischemia compromises tissue oxygenation and nutrient delivery, impairing the body's ability to mount an effective wound healing response. Furthermore, chronically elevated blood glucose levels impair immune cell function and increase the risk of microbial colonization and infection in foot ulcers. Additionally, diabetes-related microangiopathy and neuropathy contribute to poor local tissue perfusion and compromised immune responses, further exacerbating the susceptibility to infection and impeding the healing cascade [3]. Given the multifactorial nature of DFU, prompt initiation of effective management and treatment protocols is of paramount importance. Conventional therapies for DFU encompass a comprehensive approach, including wound debridement, appropriate dressings, infection management, revascularization procedures, and off-loading techniques [4]. However, despite the availability of multiple treatment options, the management of DFU remains a complex challenge, often leading to suboptimal outcomes. Consequently, there is a pressing need to explore novel therapeutic approaches that can improve the healing outcomes for DFU patients.

Over the past decades, stem cell therapy has emerged as a promising approach to address the complex pathological processes involved in diabetic wound healing. Among the various types of stem cells, mesenchymal stem cells (MSCs) have garnered significant attention due to their advantageous characteristics, including easy accessibility, lack of ethical concerns, and immunological compatibility. Numerous studies have demonstrated the potential of exogenous MSCs in protecting against local and systemic inflammatory responses associated with diabetic wounds, activating anti-apoptotic pathways, promoting tissue regeneration, and creating a favorable environment for endogenous repair [5–7]. Clinical investigations have further supported the therapeutic potential of MSC transplantation in diabetic wound healing, in terms of ulcer healing rate, improvement of lower extremity ischemia, transcutaneous oxygen pressure, and pain-free walking distance. showing accelerated functional recovery, reduced disability, and improved quality of life for patients [8–12]. Although the precise cellular and molecular mechanisms underlying the beneficial effects of MSCs in diabetic wound healing are not yet fully elucidated, it is evident that their paracrine actions play a significant role. MSCs exhibit a remarkable capacity to secrete a diverse array of growth factors, cytokines, and deposit extracellular matrix (ECM) proteins, all of which have been shown to exert profound effects on various aspects of wound healing [13–15]. Notably, the activation of the PI3K/Akt pathway is critical in this context, as it mediates the protective and regenerative effects of MSC-secreted factors. This pathway promotes cell survival, growth, and metabolism, further enhancing the therapeutic efficacy of MSCs in diabetic wound healing by facilitating tissue repair and modulating inflammatory responses [16–18]. Additionally, the application of various biomaterials, including hydrogels, has been shown to enhance the therapeutic effects of MSCs by providing a supportive environment that promotes cell retention, regulates the release of bioactive factors, and improves overall wound healing outcomes [19–21].

Despite the attractive therapeutic potential of MSCs, their clinical application has been hindered by several challenges. One major obstacle is MSCs derived from adult tissues have a limited lifespan in vitro due to replicative senescence [22, 23]. Furthermore, higher-passage MSCs are more prone to triggering immune responses upon transplantation in humans [24]. To overcome these limitations, MSCs derived from perinatal tissues, such as the placenta, umbilical cord, or amniotic fluid, offer distinct advantages. These perinatal MSCs possess multilineage differentiation potential, can be obtained through non-invasive extraction processes, have well-established immunological properties in allogeneic transplantation, are abundantly available from medical waste, and do not pose ethical concerns [25, 26]. However, despite these advantages, the use of perinatal MSCs in the treatment of DFU remains largely unexplored. Consequently, there is a significant gap in knowledge regarding the optimal selection of perinatal MSCs and their appropriate administration to maximize the benefits for DFU treatment. Moreover, there is a lack of direct comparison among different types of perinatal MSCs. In this study, we aimed to address these gaps by comparing the therapeutic effects of MSCs derived from human umbilical cord, chorionic villi, and decidua basalis of the human placenta on wound healing using db/db mice, a widely used mouse model for studying DFU. Our findings demonstrate that MSCs derived from chorionic villi and umbilical cord exert potent therapeutic effects on diabetic wound healing by enhancing re-epithelialization and angiogenesis. These results highlight the potential of these specific perinatal MSC sources as promising candidates for DFU treatment.

## Materials and methods

The work has been reported in line with the ARRIVE guidelines 2.0.

### Cell culture

Human umbilical cord mesenchymal stem cells (hUCMSCs), human decidual mesenchymal stem cells (hDCMSCs) and human chorionic villus mesenchymal stem cells (hCVMSCs) were isolated from full-term placentas obtained from three individuals. The study was approved by the Institutional Research Ethics Committee (ethical approval code: CREC 2020.313). Wharton's jelly, decidua basalis tissue, and chorionic villi tissue were dissected separately from the placentas, rinsed with PBS to remove blood, and then chopped into fine pieces (approximately 1–5 mm^3^) using sterile scissors. Segments of Wharton's jelly were directly transferred to culture flasks containing DMEM supplemented with 10% FBS and 1% penicillin/streptomycin. Cells were left undisturbed for 3–4 days and maintained at 37 °C. After attachment of Wharton's jelly fragments, the medium was changed every 2–3 days. Approximately 7–10 days later, MSCs with a fibroblastic morphology emerged. Decidua basalis and chorionic villi tissues were treated with Dispase (1 U/mL; Stemcell Technologies, Vancouver, Canada) and collagenase B (0.1%; Roche, Basel, Switzerland) for 1 h at 37 °C with gentle shaking to digest the tissues. The digested decidua basalis tissue was filtered through a 70 μm cell strainer (BD Biosciences, San Jose, CA, USA). After addition of DMEM/F12 containing 10% FBS and 1% penicillin–streptomycin to inactivate enzymes, hDCMSCs were collected by centrifugation at 340 × g for 5 min and then cultured in tissue flasks. Digested chorionic villi tissues were similarly cultured in DMEM for 7–10 days to isolate hCVMSCs. Cells were subcultured when 80–90% confluence was reached. Passages 3–8 were used in downstream applications.

The use of human bone marrow for MSC isolation was approved by Joint CUHK-NTEC Clinical Research Ethics Committee (ethical approval code: CRE-2015.018). For the adult bone marrow aspirate, MSCs were isolated by gradient centrifugation in Ficoll^®^-Paque PREMIUM 1.073 (GE Healthcare, Chicago, Illinois). The mononuclear cells were cultured in α-MEM supplemented with 10% FBS and 1% P/S as described in our previous study [27].

Human umbilical vein endothelial cells (HUVECs) and HaCaT keratinocytes (ATCC, Manassas, VA, USA) were maintained in DMEM with 10% FBS and 1% penicillin–streptomycin at 37 °C with 5% CO_2_. For experiments, cells between passages 3–10 were used.

### Flow cytometry

hMSCs were characterized using the Human MSC Analysis Kit (BD Biosciences, San Jose, CA, USA) in accordance with the manufacturer’s instructions. Briefly, the cells were detached, washed in staining buffer, and centrifuged at 200 × g for 3 min. The cell pellets were then re-suspended in the appropriate primary antibody (CD105, CD90, CD73, CD44, CD45, CD34, CD11b, CD19 and HLA-DR) at a dilution of 1:20 in staining buffer and incubated for 30 min on ice. The cells were then washed twice, re-suspended in staining buffer, and analyzed using a BD LSR Fortessa Cell Analyzer using FlowJo v10.2 Software.

### Multilineage differentiation

hMSCs at passage 3 were cultured in 6-well plates. Upon reaching 70–80% confluence, the cells were subjected to differentiation protocols to induce adipogenesis, osteogenesis, and chondrogenesis using the Adipogenesis Differentiation Kit, Osteogenesis Differentiation Kit, and Chondrogenesis Differentiation Kit (all from Gibco, Waltham, MA, USA). Following 2–3 weeks of induction, the cells underwent specific staining procedures: 0.2% Oil Red O solution (Sigma-Aldrich, St. Louis, MO, USA) for 5 min to visualize lipid accumulation in adipocytes, 2% Alizarin Red solution (Sigma-Aldrich, USA) for 20 min to detect calcium deposits in osteocytes, and 1% Toluidine Blue solution (Sigma-Aldrich, USA) for 30 min to visualize proteoglycan production in chondrocytes. The stained cells were then photographed using a Nikon Ti-2 Inverted Fluorescence Microscope to document differentiation outcomes.

### Preparation of stem cell secretome

The conditional media (CM) of hMSCs was collected according to our previous study with mild modification [28]. hMSCs were seeded at 75 cm^2^ flask and incubated in a complete culture medium. When cells reached 80–90% confluency, they were rinsed three times with PBS and replaced by 20 mL serum-free DMEM. After 48 h, the supernatant was transferred to a centrifuge tube and centrifuged at 2000 × g for 10 min to remove cell debris. The CM was concentrated and dialyzed against double-distilled water at 4 °C to remove ions and molecules below 1.0 kDa, and then freeze-dried. The protein concentration of secretome was quantified using a Pierce™ BCA Protein Assay Kit (Thermo Fisher Scientific, Waltham, MA, USA) at 562 nm.

### Cell viability assay

To assess cell viability, an MTT (3-(4,5-dimethylthiazol-2-yl)-2,5-diphenyltetrazolium bromide) assay (Sigma-Aldrich, USA) was performed as described previously [28]. hMSCs in passage 3 were seeded at a density of 1.5 × 10^3^ cells/well in 96-well plates, and their proliferation was measured daily. After adding 100 µL of fresh medium, 10 µL of MTT solution was added to each well. After 4 h of incubation at 37 °C with 5% CO_2_, the medium was replaced with 100 μL of DMSO (Santa Cruz Biotechnology, Dallas, TX, USA). The plates, covered with tinfoil, were shaken for 15 min at 100 rpm, and the absorbance at 570 nm was measured using a microplate reader. To evaluate the effects of hMSC-derived secretome on HaCaT and HUVEC proliferation, 1 × 10^3^ HaCaT cells/well or 6 × 10^2^ HUVECs/well were seeded in 96-well plates and cultured for 24 h. The culture medium was then replaced with 2% FBS-supplemented DMEM containing 5 μg/mL secretome. Cell viability was assessed daily using the MTT assay.

### Wound healing assay

HaCaT cells and HUVECs were seeded in 6-well plates. After reaching 90% confluence, a wound was created in each well using a sterile 200 μL micropipette tip. The floating cells were then removed by washing twice with PBS. The HaCaT cells and HUVECs were then subjected to DMEM containing 5 μg/mL secretome derived from three types of MSCs. Images of each wound were captured at 0 h and 24 h using a Nikon Ti-2 Inverted Fluorescence Microscope. The percentage of wound closure was then quantified using ImageJ (version 1.52a; Media Cybernetics, USA).

### Tube formation assay

The formation of capillary networks by HUVECs was evaluated using a tube formation assay on Matrigel (Corning, Corning, NY, USA). Briefly, 50 µL of Matrigel was added to each well of a cold 96-well plate and mixed thoroughly on ice. HUVECs were seeded onto the Matrigel-coated plate and incubated in culture medium for 6 h at 37 °C, with DMEM containing 5 μg/mL secretome derived from three types of MSCs. The formation of capillary-like structures was visualized using a Nikon Ti-2 Inverted Fluorescence Microscope, and the number of formed capillaries was quantified using ImageJ software.

### Transwell assay

hBMMSCs were seeded in the upper chamber of the transwell insert at a density of 1 × 10^4 cells per insert. The lower chamber was filled with DMEM containing 5 μg/mL secretome derived from three types of MSCs. Cells were allowed to migrate for 24 h at 37 °C in a humidified 5% CO_2_ incubator. After the incubation period, the cells that had migrated to the lower side of the transwell membrane were fixed with 4% paraformaldehyde and stained with crystal violet. The number of migrated cells was quantified by counting five random fields per transwell using a light microscope at 10 × magnification. Each condition was performed in triplicate, and the experiment was repeated at least three times.

### Mass spectrometry sample preparation and analysis

Secreted proteins were extracted from culture media samples using an EasyPep Mini MS Sample Prep Kit (Thermo Scientific, USA) according to the manufacturer's protocol. Briefly, 50 μg of protein from each sample was reduced with reduction solution and alkylated with alkylation solution. Excess reagents were blocked by heating and samples were digested with sequencing-grade trypsin at 37 °C for 3 h. Digests were desalted using C18 Peptide Clean-up columns. Peptides were dried using vacuum centrifugation prior to analysis.

Fractionation and LC–MS/MS analysis was performed at the University Research Facility in Chemical and Environmental Analysis, The Hong Kong Polytechnic University using an Orbitrap Fusion Lumos mass spectrometer (Thermo Fisher Scientific, USA) coupled to a Dionex Ultimate 3000 RSLCnano system. Peptides were separated using Acclaim PepMap RSLC analytical columns and a nanoEase source at a flow rate of 20 nL/min. The mass spectrometer was operated in data-dependent acquisition mode with a survey MS scan (m/z 350–1500) at a resolution of 500,000 FWHM, followed by MS/MS of the most intense precursors with charge states 2–4. Data were analyzed using Progenesis QI for proteomics (Nonlinear Dynamics, UK) and searched against the Swiss-Prot human database allowing for oxidation of methionine as a variable modification. A 1% FDR cutoff was applied at the peptide level. Differentially abundant proteins (≥ twofold, *p* ≤ 0.05) identified by ≥ 1 unique peptides across all samples were considered. The proteomics data has been deposited in MassIVE 10.25345/C5F47H56X.

#### Western blotting

**Fig. 1:**
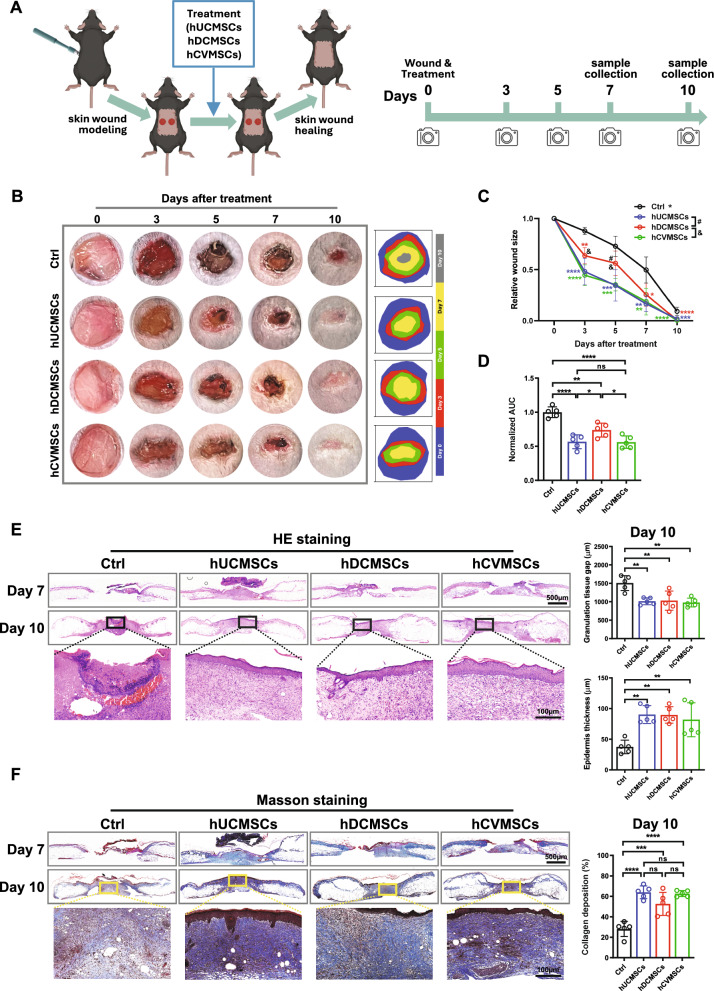
Human perinatal MSCs promote diabetic wound healing. **A** Illustration of diabetic wound healing with different hMSCs (hUCMSCs, hDCMSCs, hCVMSCs) treatment derived from perinatal tissues; **B** Representative photographs of full-thickness excision wounds at 0, 3, 5, 7 and 10 days after wounding. Dynamic traces of wound sites are shown on the right; **C** The relative wound size is shown for each group (hUCMSCs, hDCMSCs, hCVMSCs), with n = 5 for all groups. Wound areas are normalized to the original wound size and expressed as the percentage of wound closure versus initial wound size. All data are presented as mean ± SD. *, **, ***and **** represent *p* < 0.05,0.01, 0.001and 0.0001, respectively, compared to the control group. The symbol & represents *p* < 0.05 for comparisons between hCVMSCs and hDCMSCs, and the symbol # indicates *p* < 0.05 for comparisons between hUCMSCs and hDCMSCs. Statistical significance is determined using Tukey's post-hoc test following a one-way ANOVA (*p* < 0.05); **D** Mean area-under-curve (AUC) of individual wounds of each group, n = 5 for all groups. The AUC of individual wounds in the treatment groups was normalized against the mean AUC of vehicle controls within the same experimental runs. All data are presented as mean ± SD. *,**, and **** represent *p* < 0.05,0.01, and 0.0001, respectively, by Tukey’s *post-hoc* test when statistical significance by One-way ANOVA (*p* < 0.05) is obtained; **E** Representative hematoxylin and eosin (H&E) of wounds on day 7 and day 10 for each group, scale bar = 500 μm and 100 μm. Quantification of granulation tissue gap and epidermal thickness on day 10 is shown on the right, n = 5 for all groups. All data are presented as mean ± SD. **represents *p* < 0.01 by Tukey’s *post-hoc* test when statistical significance by One-way ANOVA (*p* < 0.05) is obtained; **F** Masson’s trichrome staining (MTS) of wounds on day 7 and day 10 for each group, scale bar = 500 μm and 100 μm. Quantification of collagen deposition of the wounds on day 10, n = 5 for all groups. All data are presented as mean ± SD. *** and **** represent *p* < 0.01, and 0.001, respectively, by Tukey’s *post-hoc* test when statistical significance by One-way ANOVA(*p* < 0.05) is obtained

**Fig. 2:**
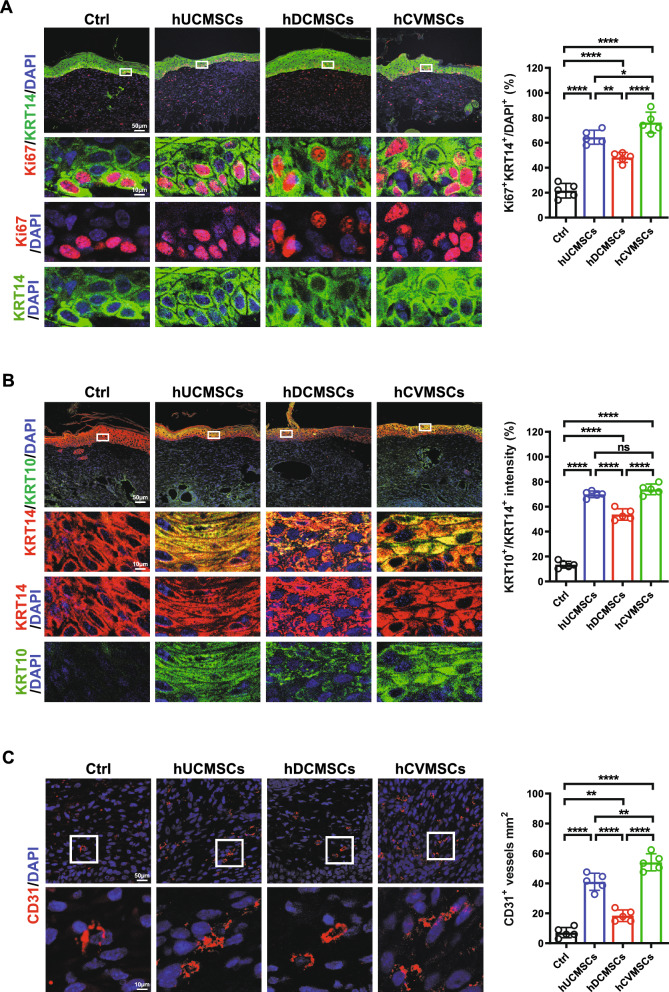
Human perinatal MSCs promote re-epithelization and angiogenesis. **A** Representative immunofluorescence (IF) images of Ki67 and KRT14 staining in wounds on day 10 for each group. Quantification of Ki67 + and KRT14 + double positive cells is shown on the right; **B** Representative IF images of KRT10 and KRT14 staining in wounds on day 10 for each experimental group. Quantification of the average intensity percentage of KRT10 + versus KRT14 + is shown on the right; **C** Representative IF images of CD31 staining in wounds on day 10 for each experimental group. Quantification of CD31 + cells per unit area is shown on the right. All data are presented as mean ± SD, n = 5 for all groups. Scale bar = 50 μm and 10 μm. *, ** and **** represent *p* < 0.05, 0.01 and 0.0001, respectively, by Tukey’s *post-hoc* test when statistical significance by One-way ANOVA (*p* < 0.05) is obtained.

**Fig. 3:**
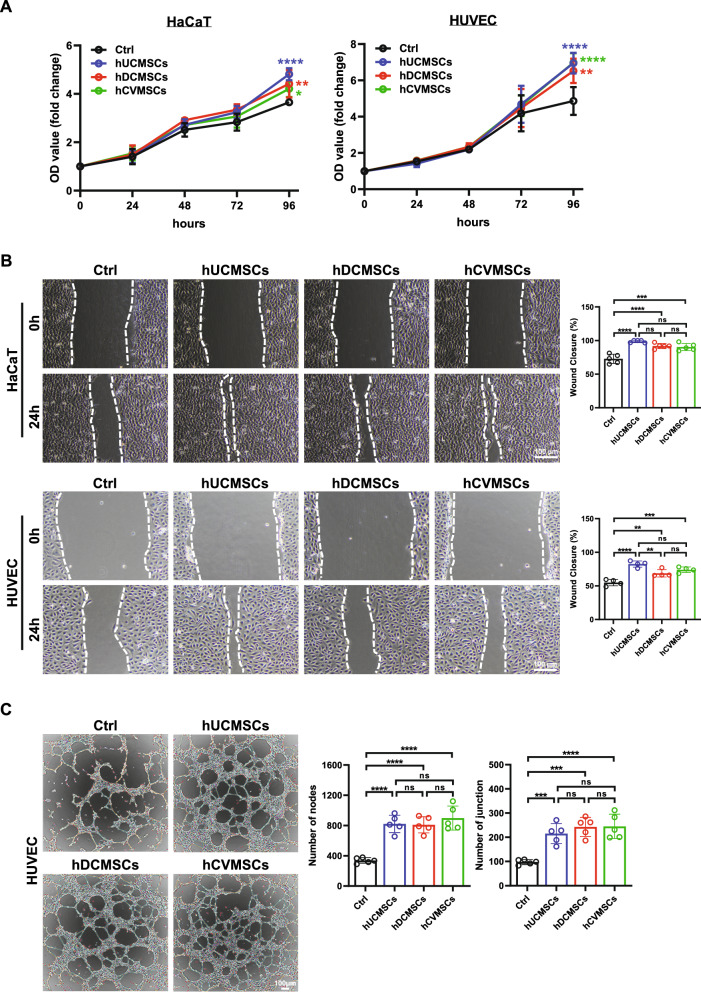
The secretome derived from human perinatal MSCs promotes proliferation, migration and angiogenesis in HaCaT and HUVECs in vitro. **A** MTT assay results showing the viability of HaCaT and HUVECs treated with secretome derived from different hMSCs, data are derived from three independent experiments; **B** Images from the wound scratch healing assay for HaCaT and HUVECs treated with secretome derived from different hMSCs, scale bar = 100 μm. Quantification of HaCaT and HUVECs migration is shown on the right. Data are derived from three independent experiments, each with triplicates; **C** Tube formation assay of HUVECs on matrigel after incubation with different secretome for 6 h, scale bar = 100 μm. Quantification of the number of nodes and junction is shown on the right. Data are derived from five independent experiments. All data are presented as mean ± SD. *, **, *** and **** represent *p* < 0.05, 0.01, 0.001 and 0.0001, respectively, by Tukey’s *post-hoc* test when statistical significance by One-way ANOVA(*p* < 0.05) is obtained

**Fig. 4:**
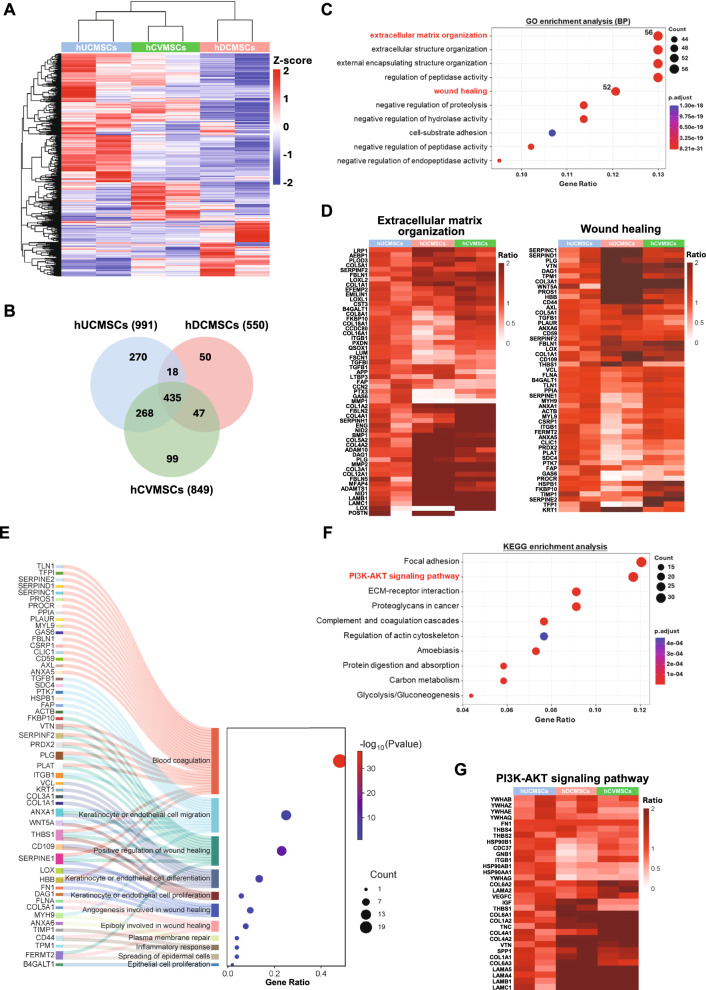
Comparative analysis of secretome profiles of perinatal MSCs. **A** Heat map showing the global protein expression profile of hUCMSCs (n = 2), hDCMSCs (n = 2) and hCVMSCs (n = 2) as determined by mass spectrometry; **B** Venn diagram illustrating the numbers of common and distinctive proteins identified by proteomics in each placental MSC group; **C** GO enrichment analysis of the 435 commonly expressed proteins; **D** Heat map analysis showing the differential expression of proteins associated with extracellular matrix organization and wound healing in the secretome of placental MSCs (compared with hUCMSCs); **E** Sankey dot plot of biological processes for the 52 commonly expressed proteins associated with wound healing; **F** KEGG pathway analysis of the 435 commonly expressed proteins; **G** Heat map analysis showing the differential expression of proteins associated with the PI3K/AKT pathway in the secretome of placental MSCs (compared with hUCMSCs)

**Fig. 5:**
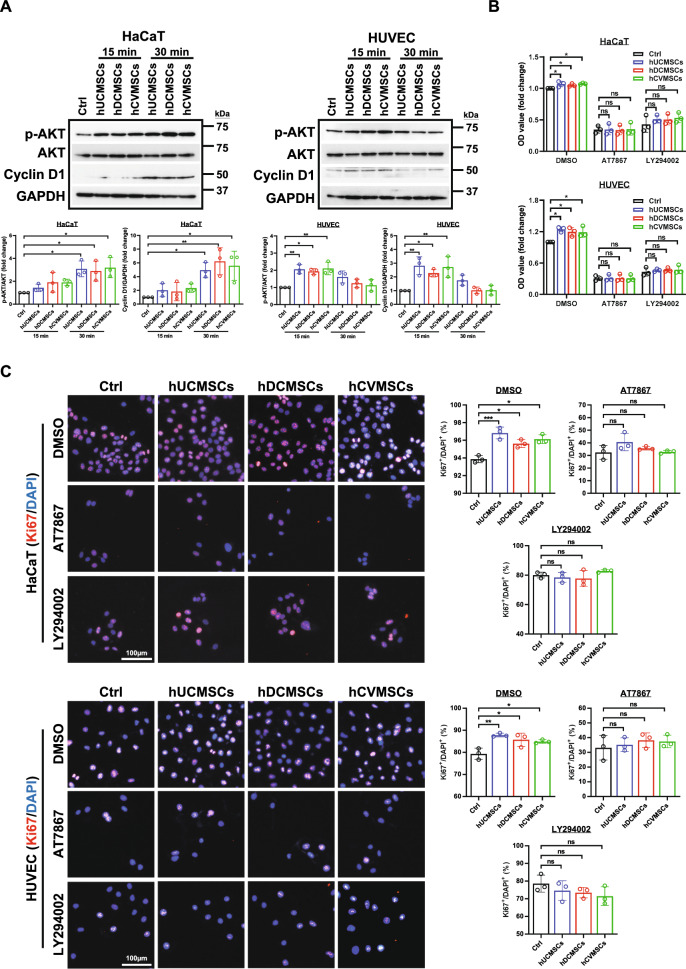
The secretome derived from perinatal MSCs promotes cell proliferation via the PI3K/AKT pathway. **A** HaCaT and HUVEC cells were treated with secretome derived from hUCMSCs, hCVMSCs, or hDCMSCs for the indicated time points. Representative western blot images and quantification showed changes in the expression levels of p-AKT and Cyclin D1. **B** Cell viability of HaCaT and HUVEC cells treated with secretome derived from hUCMSCs, hCVMSCs, or hDCMSCs in the presence or absence of the AKT inhibitor AT7867 (5 μM) or the PI3K inhibitor LY294002 (50 μM); **C** Representative immunofluorescent staining images and quantification of Ki67-positive HaCaT and HUVEC cells following 4 days of inhibitor treatment (scale bar = 100 μm). Experiments were repeated at least three times, and quantification data represented mean ± SD, *, **, and *** represent *p*<0.05, 0.01, and 0.001 by Tukey’s post-hoc test when statistical significance by One-way ANOVA(*p* < 0.05) is obtained

After exposure to different treatments, cells were lysed on ice in RIPA lysis buffer (Pierce, Rockford, IL, USA) supplemented with a protease inhibitor cocktail (Thermo Fisher Scientific, USA). Cell lysates were clarified by centrifugation at 12,000 × g for 10 min at 4 °C. Protein concentration was determined using a BCA assay kit (Thermo Fisher Scientific, USA). Proteins were separated by SDS-PAGE and transferred onto polyvinylidene difluoride (PVDF) membranes (Millipore, Billerica, MA, USA). Membranes were blocked for 1 h at room temperature in 5% non-fat milk in Tris-buffered saline containing 0.1% Tween-20 (TBST). The membranes were then incubated overnight at 4 °C with the following primary antibodies: rabbit monoclonal anti-p-AKT (1:1000, Cell Signaling Technology, Danvers, MA, USA), rabbit monoclonal anti-AKT (1:1000, Cell Signaling Technology), rabbit monoclonal anti-Cyclin D1 (1:1000, Cell Signaling Technology), rabbit monoclonal anti-VEGFR (1:500, Cell Signaling Technology), rabbit monoclonal anti-p-VEGFR2 (1:500, Cell Signaling Technology), or mouse monoclonal anti-GAPDH (1:10,000, ABclonal, China). The membranes were washed three times with TBST and incubated with the appropriate secondary antibody (1:10,000; Cell Signaling Technology) at room temperature for one hour. Protein bands were visualized using an enhanced chemiluminescence system (Bio-Rad, Hercules, CA, USA) and quantified using ImageJ software (NIH, Bethesda, MD, USA) (Figs. 1, 2, 3, 4, 5).

#### Immunofluorescence staining

**Fig. 6:**
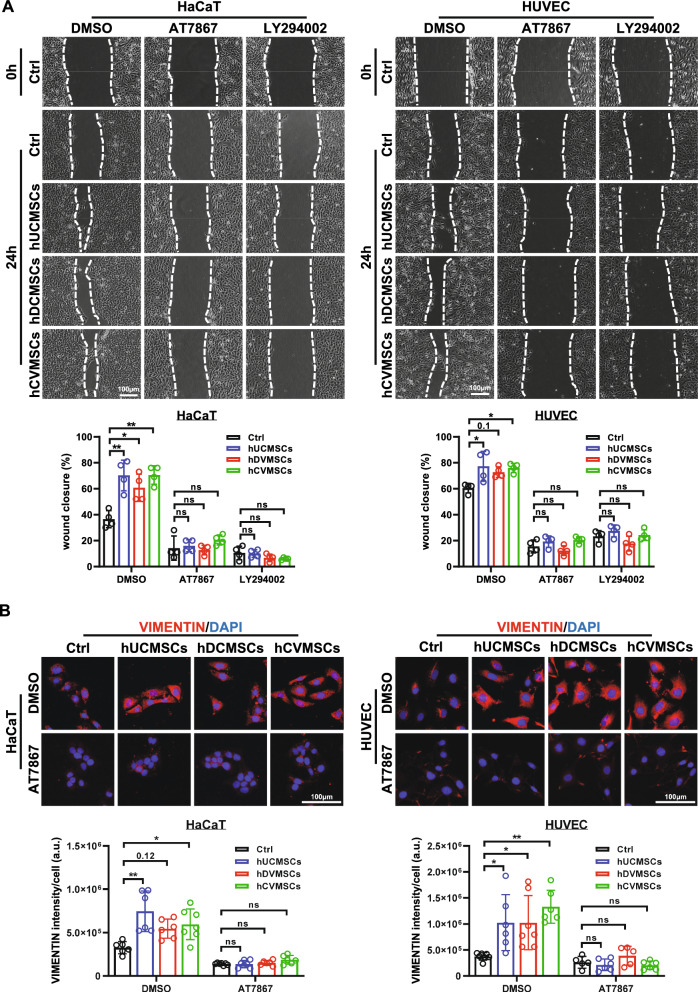
The secretome derived from perinatal MSCs promotes cell migration via the PI3K/AKT pathway. **A** Wound healing assay images and quantification of HaCaT and HUVEC cells cultured in media supplemented with secretome derived from hUCMSCs, hCVMSCs, or hDCMSCs in the presence or absence of the inhibitors; **B** Representative immunofluorescent staining images and quantification of the mean Vimentin intensity in HaCaT and HUVEC cells following one day of inhibitor treatment (scale bar = 100 μm). Experiments were repeated at least three times, and quantification data represented mean ± SD, * and ** represent *p*0.05 and 0.01 by Tukey’s post-hoc test when statistical significance by One-way ANOVA(*p* < 0.05) is obtained

Both HaCaT and HUVEU cells were cultured on cover slip to analyse their proliferative and migrative capacities, with or without secretome treatment by IF staining with Ki67 (Fig. 5C) or Vimentin (Fig. 6B). Cultured cells grown on coverslips were fixed in 4% paraformaldehyde (PFA, Sigma–Aldrich, USA) for 20 min at room temperature and washed three times with PBS. Cells were permeabilized with PBS containing 3% BSA and 0.3% Triton X-100 (Santa Cruz, USA) for 10 min at room temperature. After blocking with 3% BSA, the fixed cells were incubated with the appropriate primary antibodies at 4 °C overnight. Cells were incubated with primary antibodies against Ki67 (1:200, rat monoclonal, Invitrogen, Waltham, MA, USA) and vimentin (1:50, goat polyclonal, Santa Cruz, USA) overnight at 4 °C. After washing, cells were incubated with Alexa Fluor 594-conjugated anti-rat and Alexa Fluor 594-conjugated anti-goat secondary antibodies (1:400, Invitrogen, USA) for 1 h at room temperature in the dark. Nuclei were stained with 4',6-diamidino-2-phenylindole (DAPI, 1 μg/mL, Sigma-Aldrich) for 5 min. Coverslips were mounted onto slides using fluorescence mounting medium (Dako, Glostrup, Denmark). For cell line studies, the percentages of positive cells were calculated. The percentage of positive cells was defined as the number of positive cells / DAPI positive cells. Five fields were analyzed per slide under the microscope and no less than 200 cells were analyzed for quantification data. All experiments were conducted three times, and each experiment was set up with triplicate samples.

#### Preparation of PEGDA/SA/Col-I hydrogel

Sodium alginate powders derived from brown algae (SA, medium viscosity, Sigma, USA) were sterilized by ultraviolet (UV) light before they were dissolved in PBS to produce 2% SA solution (w/v). The SA solution was further sterilized with a 0.22 μm filter (Millipore) before use. Poly (ethylene glycol) diacrylate (PEGDA, 1 kDa, EFL, China) was dissolved in LAP solution (Lithium Phenyl (2,4,6-trimethylbenzoyl) phosphinate, 0.2% w/v in PBS) to produce 12% PEGDA solution (w/v). The PEGDA solution was further sterilized with a 0.22 μm filter (Millipore) before use. Collagen I (Col I, Corning, USA) was diluted by PBS at the ratio of 1:20 before use.

The hydrogel mixture was prepared by mixing 100 μL SA, 100 μL PEGDA solution, and 20 μL Col I through a t-branch pipe, and then exposed to UV irradiation (λ = 365 nm) for 60 s. After UV crosslinking, 100 mM CaCl_2_ solution was applied for secondary crosslinking for 60 s. After rinsing with PBS, the PEGDA/SA/Col-I hydrogel was obtained. For comparison, 2% SA solution was crosslinked by 100 mM CaCl_2_ and 6% and 12% PEGDA were crosslinked by UV irradiation (λ = 365 nm) for 60 s, respectively, as control.

#### Swelling ability

The swelling ratio (%) of each hydrogel was measured via monitoring the change in weight during the swelling process. Briefly, prior to swelling, 0.2 mL of hydrogel was formed in a cylindrical mold, and weighed as *W*_o_ (g), then immersed into a 15 mL of centrifuge tube filled with deionized water or PBS at 25 °C and taken out at a scheduled time interval. After the excess water was removed with absorbent paper, the hydrogels were separately weighed as *W*_t_ (g). The swelling ratio of the hydrogels were calculated by the following formula. $\text{Swelling ratio }\left(\text{\%}\right)={(W}_{\text{t}}/{W}_{\text{o}})\times 100\%$

#### Compression modulus

For compressive tests, the hydrogels with a height of 5 mm and a diameter of 10 mm were fabricated. The compressive test was conducted with 50% strain at a rate of 30 mm min^−1^ using the Mach-1 Mechanical Tester Model (Biomomentum, CAN). The compressive modulus was determined from the slope of the linear regression in the 50–60% strain range.

#### Fabrication of hMSC-laden hydrogel

The PEGDA solution, SA solution, collagen I solution, and hMSCs suspension (1 × 10^8^ cells/mL) were mixed evenly at a volume ratio of 100:100:1:20, resulting in a final hMSC concentration of approximately 1 × 10^7^ cells/mL.

Then, 100 μL of the mixed gel solution was added to each well of a 6-well plate and exposed to UV irradiation (365 nm, 10 mW/cm^2^) for 1 min to cross-link the PEGDA. Subsequently, 1 mL of 100 mM calcium chloride solution was added and allowed to react for 1 min to cross-link the SA. Finally, the hydrogel was washed three times with PBS to obtain a PEGDA/SA/Col-I hydrogel encapsulating hMSCs. To assess cell viability, 5 μL of the MSCs-laden mixed gel solution was added to each well of a 96-well plate. Following cross-linking and washing, 100 μL of complete culture medium was added, and the samples were incubated for 7 days. Cell proliferation was evaluated by MTS on days 0, 4, and 7 of the culture period.

#### Live/Dead assay

Cell viability was assessed in hMSCs-laden PEGDA/SA/Col-I 1 and 3 days post-culture using a Live/Dead Viability/Cytotoxicity Kit (Invitrogen, USA). Hydrogels were washed 3 times in PBS, then incubated with 2 μM calcein AM and 4 μM EthD-1 for 30 min at room temperature. Stained hydrogels were imaged using a fluorescence microscope (Nikon Ti-2).

#### Secreted protein assay

100 μL of hMSCs-laden SA/PEGDA/Col-I hydrogel was plated per well in 6-well plates and incubated for 24 h. The culture medium was then removed from each well and the cell-laden hydrogels were washed three times with serum-free DMEM. Subsequently, 2 mL of serum-free DMEM was added to each well. After 48 h, medium was collected, centrifuged at 2000 × g for 10 min to pellet debris, and secreted protein quantified using a Pierce BCA Protein Assay Kit at 562 nm.

#### H_2_O_2_ or high-glucose stress induction

5 μL of hMSCs-laden PEGDA/SA/Col-1 hydrogel or 5 × 10^3^ hMSCs were plated per well in 96-well plates and incubated for 24 h prior to the experiment. Subsequently, experimental groups were treated with 300 mM H_2_O_2_ for 2 h or 200 mM glucose for 24 h. The supernatant of each well was then removed and replaced with 100 µL of fresh medium containing 10 µL of MTS reagent (CellTiter 96^®^ AQueous One Solution Cell Proliferation Assay, Promega, Madison, WI, USA). After 4-h incubation period, 100 µL of solution per well was transferred to a new 96-well plate for absorbance measurement at 490 nm.

#### Cutaneous wound healing model in db/db mice and treatment strategy

All animal use and research protocols in this study were approved by the Laboratory Animal Experimentation Ethical Committee, Chinese University of Hong Kong, and were in accordance with the Guideline for the Care and Use of Laboratory Animals. All male db/db mice (6–8 weeks old) were obtained from the Chinese University of Hong Kong Laboratory Animal Service Center and housed in a barrier environment (22 °C, 45% humidity, and a 12/12 light/dark cycle). To create full-thickness wounds, 60 db/db mice were anesthetized using 2% isoflurane, and two circular 6 mm or 8 mm biopsy punches (Integra Miltex) were applied to their dorsum. The db/db mice typically exhibit significantly elevated blood glucose concentrations, often exceeding 14 mmol/dL 6 h after feeding, which models type 2 diabetes and its complications. Mice were randomly assigned to experimental groups, after inflicting skin wounds. Sample size determination was performed using G*Power 3.1 [29]. Effect size for wound healing analyses was determined on the basis of pilot experiments, and calculations were made on the basis of the following criteria: power of 80% and a significance level < 0.05. Sample sizes for each experiment are included in the figure legends. The researchers involved in biochemical analysis were blinded to group allocations for the duration of the study.

To assess the therapeutic effects of three different types of hMSCs on wound healing with a diameter of 6 mm full-thickness skin wounds, 30 db/db mice were randomly divided into groups receiving (n = 5 per group per tissue collection time point): (1) vehicle (20 µL PBS) and hUCMSCs (1 × 10⁶ cells in 20 µL PBS), (2) vehicle (20 µL PBS) and hDCMSCs (1 × 10⁶ cells in 20 µL PBS), and (3) vehicle (20 µL PBS) and hCVMSCs (1 × 10⁶ cells in 20 µL PBS) applied topically to each wound. Skin tissues were collected at day 7 and day 10 for characterization.

To assess the effect of hMSCs-laden PEGDA/SA/Col-I hydrogels with a diameter of 8 mm full-thickness skin wounds. To ensure the PEGDA/SA/Col-I hydrogel remains securely adhered to the wound despite mouse movement, we prepared sterile hydrogels that matched the size of the wound. After gently placing the hydrogels onto the wound, we covered them with dressings. The dressings not only help keep the hydrogels in place but also reduce the risk of infection. 30 db/db mice were assigned to groups receiving (n = 5 per group per tissue collection time point): (1) vehicle (20 µL PBS) and cell-free PEGDA/SA/Col-1 hydrogel (100 µL), (2) hUCMSCs (1 × 10⁶ cells in 20 µL PBS) and hUCMSCs-laden PEGDA/SA/Col-1 hydrogel (1 × 10⁶ cells in 100 µL), and 3) hCVMSCs (1 × 10⁶ cells in 20 µL PBS) and hCVMSCs-laden PEGDA/SA/Col-1 hydrogel (1 × 10⁶ cells in 100 µL). PBS or hMSCs were applied topically to each wound, whereas hydrogels were plated directly onto the wound. Skin tissues were collected on day 7 and day 11 for characterization. The wound area was measured on days 0, 3, 5, 7 and 10 or 0, 3, 5, 7, 9 and 11 using a 30-cm ruler and a silicone template with a 6 mm or 8 mm hole. Images were then analyzed using ImageJ software. The wound areas of each wound were calculated by the following formula: Relative wound size = (original wound area − final wound area) / original wound area. Normalized area under the curve (AUC) = AUC of treatment group/mean AUC of vehicle controls.

#### Histology, immunohistochemistry and immunofluorescence

Full layers of back skin were harvested on days 7 and 10 or 11. For this purpose, animals were euthanized. Ketamine-xylazine combination (300 mg/kg + 30 mg/kg) was administered intraperitoneally for animal euthanasia. Then, the harvested tissues were fixed in 4% paraformaldehyde and embedded in paraffin. Masson's trichrome staining, hematoxylin and eosin (H&E) staining, and immunofluorescence (IF) were performed on 5 µm sections. For IF, antigen retrieval was performed using citrate buffer (10 mM, 0.05% Tween 20, pH 6.0) in a microwave at high power for 3 min and then in a steamer at 95 °C for 15 min. The following primary antibodies were used for IF: rabbit monoclonal antibody (mAb) to KRT14 (1:200, Proteintech, Rosemont, IL, USA), mouse mAb to KRT10 (1:200, Invitrogen, USA), rat mAb CD31 (1:200, Abcam, Cambridge,UK), and rat mAb Ki67 (1:200, Invitrogen, USA). Sections were first blocked for 1 h at room temperature and then incubated with primary antibodies at 4 °C overnight. Secondary antibodies were incubated for 1 h at room temperature. Alexa Fluor 568 donkey anti-rabbit IgG (1:400, Invitrogen, USA), Alexa Fluor 488 donkey anti-mouse IgG (1:400, Invitrogen, USA), and Alexa Fluor 594 donkey anti-rat IgG (1:400, Invitrogen, USA) were used. DAPI was used to counterstain nuclei. Images were captured using a confocal system with an inverted microscope (Leica TCS SP8 Inverted Confocal Microscope) and analyzed with Image J software. For all quantitative analyses, images were collected using the same acquisition parameters to facilitate fluorescence intensity comparisons between groups. For all studies, a region of interest (ROI) was chosen based on the anatomical structures of the skin based on DAPI staining. Spots were designated as positive cells when their signals were above a set threshold signal intensity; and this threshold was applied across all analyses. Specifically, for measuring epidermal thickness, we utilized ImageJ to measure the thickness at three positions—left, middle, and right—of the epidermis and averaged these values for comparison across groups. To assess the granulation tissue gap, we employed ImageJ’s freehand tool to measure the size of the granulation gap, allowing for comparative analysis between groups. For collagen deposition analysis, images were processed using ImageJ (Fiji) to perform three-color separation, isolating the collagen, which appears as a darker blue stain. We quantified the collagen deposition by measuring the area of collagen-positive staining relative to the total tissue area.

#### Statistical analysis

Statistical analyses were performed using Prism V6.01 (GraphPad Software). For comparisons between two groups with normally distributed data, a two-tailed Student's t-test was employed. For comparisons involving more than two groups, one-way analysis of variance (ANOVA) with Tukey's multiple comparison post hoc test was used. When two independent variables were assessed, two-way ANOVA with Tukey's post hoc test was applied. Post hoc test p-values are presented only when an overall statistically significant difference was observed after ANOVA. Data are presented as mean ± SD. A *p*-value less than 0.05 was considered statistically significant.

## Results

### Mesenchymal stem cells from perinatal tissues promote diabetic wound healing

A total of nine cell lines were established, with three lines each for hCVMSCs, hDCMSCs, and hUCMSCs, as described in the Materials and Methods section. Similar morphological characteristics were observed among the three types of MSCs (Fig. S1A). To assess their proliferative capacities, MTT analysis was conducted at the 3rd passage, revealing that hUCMSCs exhibited a higher proliferative capacity compared to hDCMSCs and hCVMSCs (Fig. S1B). Flow cytometry analyses demonstrated that more than 95% of all three MSC types expressed CD105, CD44, and CD73, while being negative for hematopoietic markers CD34, CD11, CD19, CD45, and HLA-DR (Fig. S2A). A minor difference was observed in CD90 expression, with approximately 85.6% of hCVMSCs expressing CD90, while approximately 97.5% of hDCMSCs and 100% of hUCMSCs expressed CD90 (Fig. S2A). Additionally, all three types of MSCs demonstrated the ability to differentiate into osteogenic, adipogenic, and chondrogenic lineages, as confirmed by Alizarin red, Oil red O, and Alcian blue staining, respectively (Fig. S2B).

To evaluate the therapeutic potential of different types of MSCs in diabetic wound healing, we conducted experiments using db/db mice, a well-established model for type II diabetes. Full-thickness skin wounds with a diameter of 6 mm were created on the dorsal surfaces of the db/db mice. Subsequently, the wounds were treated with PBS (vehicle), 1 × 10^6^ hUCMSCs, hDCMSCs, or hCVMSCs on the same day as the skin injury. The progression of wound healing was monitored by imaging the wounds at 2–3-day intervals until complete closure (Fig. 1A). As depicted in Fig. 1B, noticeable differences in wound healing rates were observed between the vehicle and MSC-treated groups, starting from Day 3. All MSC-treated groups exhibited accelerated reduction in wound size compared to the vehicle group. Notably, both hUCMSCs and hCVMSCs groups displayed more efficient wound-healing kinetics, demonstrating significant differences when compared to the hDCMSCs group from Day 3 to Day 5 (Fig. 1C). However, by Day 10, all three MSC-treated groups showed similar wound closure rates, with nearly complete closure, while the average wound size of the control group was 9.2 ± 4.0% (Fig. 1C). To further analyze the wound healing outcomes, the area-under-curve (AUC) of each wound was calculated and normalized as average values against the vehicle group. The normalized AUCs of the hUCMSCs and hCVMSCs groups were significantly lower than those of the hDCMSCs group (Fig. 1D). In line with the observed rates of wound closure, histological analysis conducted on Day 10 after stem cell treatment revealed a notable decrease in the granulation tissue gap and increase in the thickness of the newly formed epidermal layer in all stem cell-treated groups, displaying more efficient re-epithelialization and well-formed epidermis when compared to the vehicle group (Fig. 1E). No discernible differences were observed among the three stem cell groups. Furthermore, Masson's trichrome (MST) staining demonstrated increased collagen deposition in the stem cell-treated groups (Fig. 1F). In conclusion, our findings demonstrate that MSCs derived from umbilical cord, chorionic villi, or decidua basalis have a significant potential to promote diabetic wound healing. Among the three types, hDCMSCs exhibited a slightly weaker ability.

### Mesenchymal stem cells from perinatal tissues promote diabetic wound healing by facilitating re-epithelization and angiogenesis

Wound healing in adult mammals involves a complex series of events, including inflammation, re-epithelialization through keratinocyte migration and proliferation, granulation tissue formation, and neovascularization. These processes are interconnected and crucial for successful wound closure [30]. Re-epithelialization is a critical step in wound closure, involving the migration, proliferation, and differentiation of epidermal keratinocytes [31]. To evaluate the effects MSCs on keratinocyte behavior during wound healing, we used Keratin 14 (KRT 14) and Keratin 10 (KRT 10) to assess immature and mature keratinocytes in the basal and suprabasal layers, respectively during wound healing. Our results revealed a remarkable increase in proliferating KRT 14^+^ cells (KRT14^+^/Ki67^+^) in the MSC-treated groups compared to the vehicle group on Day 10. Notably, the number of double-positive cells was significantly lower in the hDCMSCs group compared to the hUCMSCs or hCVMSCs groups (Fig. 2A). Additionally, the expression levels of KRT10 were significantly higher in the MSC-treated groups compared to the vehicle group. Furthermore, the intensity ratios between KRT10 and KRT14, indicating keratinocyte maturation, were significantly increased in the MSC-treated groups. Of note, the hDCMSCs-treated group exhibited a significantly lower ratio compared to both the hUCMSCs and hCVMSCs groups (Fig. 2B). Angiogenesis plays a crucial role in wound healing by providing oxygen and nutrients to the wound area. Notably, enhanced vascularization was observed in the wounds treated with MSCs, as evidenced by the higher density of CD31^+^ vessels compared to the vehicle group at Day 10 (Fig. 2C). Similarly, the pro-angiogenic effect was more pronounced in the hUCMSCs and hCVMSCs groups compared to the hDCMSCs group. Collectively, our results demonstrate that MSCs derived from umbilical cord, chorionic villi, or decidua basalis effectively promote re-epithelialization and angiogenesis during diabetic wound healing.

### The secretome derived from perinatal MSCs exhibits a multifaceted regulatory impact on various cell functions in vitro.

It is well-established that the therapeutic efficacy of MSCs primarily stems from their trophic effects, which promote endogenous repair mechanisms. To assess the regulatory effects of secretory factors derived from perinatal MSCs on different cell types involved in diabetic wound healing, we collected secretome from the three MSC types. Initially, we examined the impact of the secretome on cell viability and observed enhanced cell viability in both the keratinocyte cell line, HaCaT, and the endothelial cell line, HUVEC. No significant differences were observed among the secretome derived from the three MSC types (Fig. 3A). Subsequently, we evaluated the effect of the secretome on cell migration using a scratch wound assay. The results revealed that the secretome markedly enhanced cell migration in HaCaT, HUVEC, and Mouse Embryonic Fibroblasts (MEFs) (Figs. 3B, S3A). Notably, hUCMSCs exhibited a superior effect on promoting endothelial migration compared to hDCMSCs (Fig. 3B). Furthermore, we conducted a tube formation assay to assess capillary network formation in HUVECs. The results demonstrated that the secretome groups significantly improved the number and nodes of junctions compared to the control group, indicating their advantage in promoting angiogenesis (Fig. 3C). Lastly, we investigated the effect of the secretome on the recruitment of human bone marrow MSCs (hBMMSCs), which are known to play a role in skin regeneration during wound healing[32, 33], using a transwell assay. The results revealed that the secretome groups exhibited a remarkable ability to recruit hBMMSCs (Fig. S3B). Taken together, these findings collectively indicate that perinatal MSCs promote diabetic wound healing through the regulatory effects of their secretory factors on multiple aspects of cell functions. It is also important to note that while some differences were observed among the secretome derived from different types of MSCs, they all share common features, such as enhancing cell viability, promoting cell migration, facilitating angiogenesis, and recruiting hBMMSCs, that contribute to their therapeutic effects.

### Comparative analysis of secretome profiles of perinatal MSCs reveals biological pathways associated with wound healing

While the three types of MSCs used in this study were derived from perinatal tissues, a direct comparison of their secretory factors has not been previously reported. Therefore, we utilized mass spectrometry to analyze the protein components in the secretome derived from hUCMSCs, hCVMSCs, and hDCMSCs, followed by unsupervised principal component analysis (PCA). A total of 1187 proteins were identified in the secretome, with quantitative information available for 1080 proteins. PCA results revealed distinct clusters corresponding to the three types of MSCs (Fig. S4A), with the secretory profiles of hCVMSCs exhibiting greater similarity to hUCMSCs compared to hDCMSCs (Fig. 4A, B). Among the 1187 proteins, 435 were commonly detected in all three MSC types (Fig. 4B). Gene ontology (GO) analysis of the common proteins demonstrated their involvement in extracellular matrix (ECM) organization, extracellular structure organization, regulation of peptidase activity, and wound healing (Fig. 4C, D), highlighting the enrichment of ECM regulatory proteins that relate to wound healing. To further explore the potential molecular mechanisms underlying the therapeutic effects of MSCs, we analyzed proteins specifically associated with wound healing (n = 52). The results revealed groups of proteins involved in blood coagulation, keratinocyte or endothelial cell migration, differentiation and proliferation, angiogenesis, plasma membrane repair, inflammatory response and spreading of epidermal cells (Fig. 4E). Interestingly, proteins such as THBS1 (thrombospondin 1), SERPINE1 (serpin family E member 1), ANXA1 (annexin A1), LOX (lysyl oxidase), and ITGB1 (integrin beta-1) have been implicated in multiple cellular functions during wound healing. Notably, there were proteins exclusively detectable in hUCMSCs, hDCMSCs, or hCVMSCs, respectively (Fig. S4B). For example, proteins exclusively detected in hUCMSCs were involved in functional categories related to the regulation of translation and amino acid metabolic processes. In contrast, proteins more enriched in hCVMSCs were involved in ECM organization and immune response, while proteins enriched in hDCMSCs were involved in blood coagulation and tissue homeostasis (Fig. S5A, B). KEGG pathway mapping of the common proteins showed that the PI3K/AKT signaling pathway is markedly regulated by all three types of MSCs (*n* = 32, 7.36%, Fig. 4F). Noteworthy proteins involved in the activation of the PI3K/AKT pathway included ECM proteins such as collagen IV, collagen VI, fibronectin (FN1), laminin, and THBS1. Additionally, various signaling proteins, including secreted phosphoprotein 1 (SPP1), heat shock protein 90 beta family member 1 (HSP90B1), vascular endothelial growth factor C (VEGFC), and insulin-like growth factor 2 (IGF2), were also detected as potential mediators of the signal transduction (Fig. 4G).

### The secretome derived from perinatal MSCs enhances cell proliferation and migration in HaCaT and HUVECs via activation of the PI3K/AKT pathway

The PI3K/AKT pathway plays a crucial role in various cellular processes involved in wound healing, including cell proliferation, migration, and angiogenesis [9]. To investigate the involvement of the PI3K/AKT pathway in the regulatory effects of MSCs on wound healing, we evaluated the activation of PI3K/AKT signaling in HaCaT and HUVEC cells treated with secretome derived from hUCMSCs, hDCMSCs, and hCVMSCs (Fig. 5A). Our results demonstrated that treatment with the secretome significantly increased AKT phosphorylation in both HaCaT cells (at 30 min) and HUVECs (at 15 min), indicating activation of the PI3K/AKT pathway. Moreover, the secretome treatment also upregulated Cyclin D1 expression in both cell types, which was consistent with the activation of the PI3K/AKT pathway. Next, we examined the impact of suppressing the PI3K/AKT pathway on secretome-enhanced cell proliferation using MTT assay and Ki67 staining. After 4 days of culture, we observed increased proliferation in both HaCaT cells and HUVECs exposed to the secretome compared to the control group. However, this enhanced cell proliferation was completely abolished when the PI3K/AKT pathway was suppressed by LY294002 or AT7867 treatment (Fig. 5B, C). In accordance with these in vitro data, treatment with the secretome derived from three types of hMSCs significantly activated VEGFR2 signaling in endothelial cells (Fig. S6), which is highly associated with the PI3K/AKT pathway in the wound healing process [34].

To further investigate the underlying mechanisms by which the MSC secretome promote wound healing, we performed in vitro scratch wound healing assays. The results showed that the secretome from all three MSC types significantly enhanced the migratory capacity of both HaCaT and HUVEC cells compared to the control group (Fig. 6A). Interestingly, when the cells were co-treated with the secretome and specific inhibitors of the PI3K/AKT pathway, either LY294002 or AT7867, the pro-migratory effects of the secretome were completely abolished. Furthermore, immunostaining analysis revealed that the secretome induced the expression of Vimentin, a well-known marker of cell migration during wound healing. Importantly, this Vimentin upregulation was completely suppressed by the AKT inhibitor AT7867 (Fig. 6B). Collectively, these findings indicate that the secretome derived from hUCMSCs, hDCMSCs, and hCVMSCs stimulates the proliferation and migration of HaCaT cells and HUVECs through the activation of the PI3K/AKT pathway.

### Hydrogel encapsulation enhances MSCs survival and secretion in vitro

Hydrogel encapsulation has gained significant attention in maximizing the therapeutic potential of stem cells for wound healing [35]. With their three-dimensional network resembling the ECM, hydrogels offer a protective environment for stem cells within the inflammatory microenvironment of diabetic wounds, safeguarding their viability against reactive oxygen species (ROS) in wound exudate. Alginate (SA) hydrogels have emerged as a promising approach for protecting allogenic MSCs from host defense mechanisms, providing immunoprotection and prolonged cell survival. However, achieving optimal cell viability and functionality within alginate gels remains a challenge, necessitating further optimization of gel properties and incorporation of bioactive cues to enhance therapeutic efficacy [36].

**Fig. 7:**
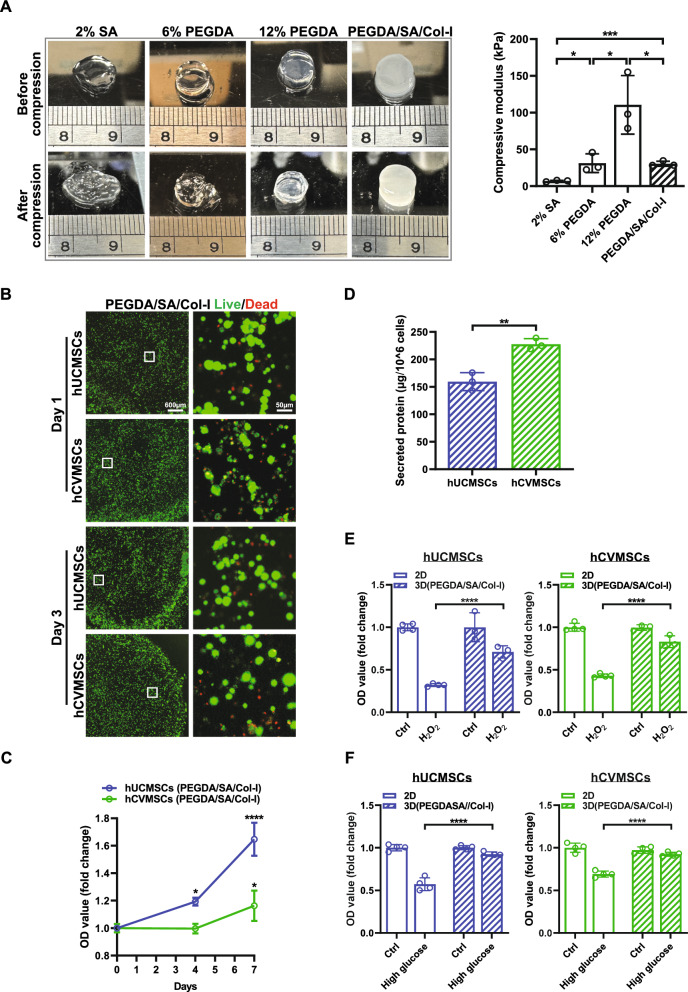
PEGDA/SA/Col-I hydrogel encapsulation enhances MSCs survival and secretion in vitro. **A** Measurement of the compressive modulus of the hydrogels. The hydrogels were all compressed by 50% of height to measure the compressive modulus. Images on the left show that after compression, the PEGDA/SA/Col-I hydrogel retained its shape the best among all hydrogels tested, suggesting its potential as an elastic wound dressing; **B** Live/Dead fluorescent images of hUCMSCs and hCVMSCs cultured within PEGDA/SA/Col-I hydrogel for 1 and 3 days (scale bar = 600 μm); **C** MTS assay results determining the cell viability of hUCMSCs and hCVMSCs encapsulated in the PEGDA/SA/Col-I hydrogel; **D** Quantification of the total secreted protein content from MSCs-laden PEGDA/SA/Col-I hydrogel; **E** hUCMSCs and hCVMSCs cultured in 2D or encapsulated in 3D PEGDA/SA/Col-I hydrogel were treated with H2O2 (300 mM) for 2 h, and cell viability was assessed by MTS assay; **F** hUCMSCs and hCVMSCs cultured in 2D or encapsulated in 3D PEGDA/SA/Col-I hydrogel were treated with high-glucose (200 mM) for 24 h, and cell viability was assessed by MTS assay. Experiments were repeated at least three times, and quantification data represented mean ± SD. *, **, *** and **** represent *p* < 0.05,0.01, 0.001, and 0.0001 by Student Ttest or One-way ANOVA(*p* < 0.05)

In this study, we aimed to enhance the therapeutic effects of MSCs on wound healing by incorporating polyethylene glycol diacrylate (PEGDA) and collagen I (Col-I) into SA hydrogel, leveraging their synergistic effect. Initially, we compared the physical properties of SA, PEGDA, and PEGDA/SA/Col-I hydrogels. As shown in Fig. 7A, the PEGDA/SA/Col-I hydrogel exhibited a compressive modulus similar to that of the 6% PEGDA hydrogel, which was higher than that of the SA hydrogel but lower than that of the 12% PEGDA hydrogel. This intermediate compressive modulus is advantageous for applications requiring both structural integrity and conformity to irregular surfaces. Notably, the PEGDA/SA/Col-I hydrogel retained its shape the best after compression, indicating its potential as an elastic wound dressing. We also evaluated the swelling capacity of the different hydrogels in H_2_O and PBS. The SA hydrogel exhibited high swelling ratios (~ 250%) in both H_2_O and PBS. In comparison, the PEGDA/SA/Col-I hydrogel demonstrated favorable swelling ratios (150–220%) relative to the 12% PEGDA hydrogel (Fig. S7). Overall, these results demonstrate that the PEGDA/SA/Col-I hydrogel possesses a high swelling ratio and good structural integrity after deformation, which are critical properties for absorbing excessive exudate while maintaining its structure during wound repair.

Considering that the PEGDA/SA/Col-I hydrogel provides a biologically relevant scaffold resembling the native ECM, we conducted in vitro experiments to determine its regulatory effects on the protection and secretion activities of hUCMSCs and hCVMSCs. The results showed excellent biocompatibility of the PEGDA/SA/Col-I hydrogel, with limited cell death observed (Fig. 7B). Moreover, both hUCMSCs and hCVMSCs exhibited continued growth within the hydrogel (Fig. 7C). Subsequently, we evaluated the quantity of proteins secreted by MSCs and found that MSCs encapsulated in SA/PEGDA/Col-I hydrogel secreted proteins efficiently, reaching approximately 150–200 µg/10^6 cells (Fig. 7D). To further assess the protective role of the PEGDA/ SA/Col-I hydrogel, we exposed MSCs to H_2_O_2_ or high glucose, inducing ROS accumulation that mirrors the hostile environment observed in diabetic wounds. We compared the survival rates of MSCs encapsulated in the hydrogel to those cultured in a traditional 2D culture system. The results revealed significantly higher survival rates for hUCMSCs and hCVMSCs encapsulated in the hydrogel compared to cells in the 2D culture (Fig. 7E, F). These findings highlight the ability of the PEGDA/SA/Col-I hydrogel to provide a favorable microenvironment for enhancing MSC survival, even under harsh conditions.

### MSC-laden hydrogel exhibits enhanced diabetic wound healing effects

**Fig. 8:**
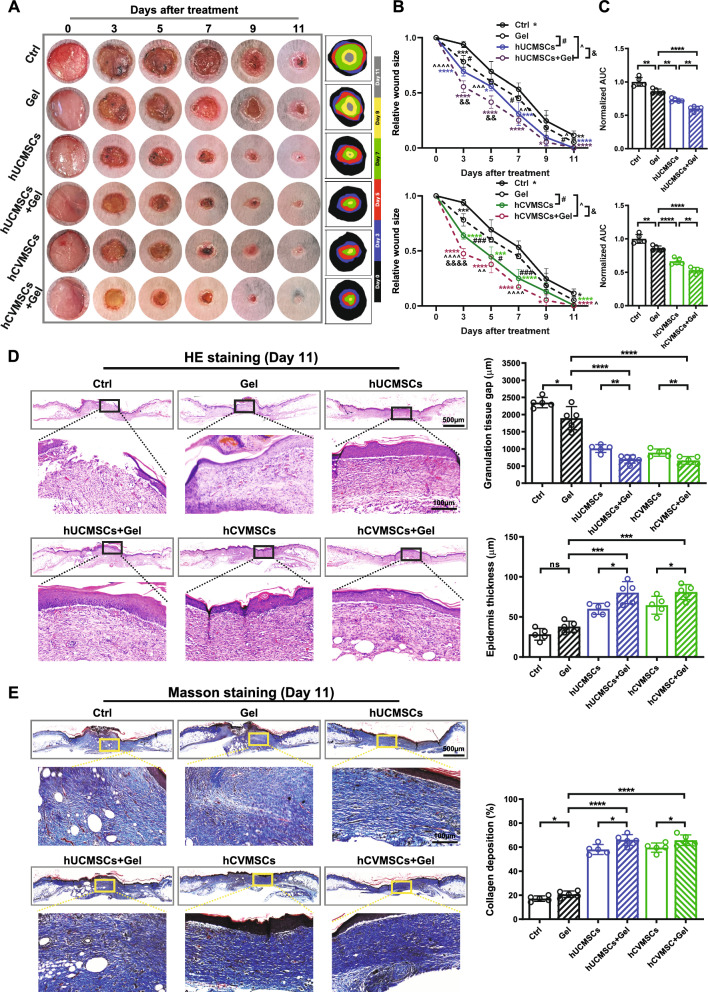
PEGDA/SA/Col-I hydrogel encapsulation enhances MSC therapeutic efficacy for diabetic wound healing. **A** Representative photographs of full-thickness excision wounds at 0, 3, 5, 7, 9 and 11 days after wounding. Dynamic traces of wound sites are shown on the right; **B** The relative wound size is shown for each group, with n = 5 for all groups. Wound areas are normalized to the original wound size and expressed as the percentage of wound closure versus initial wound size. All data are presented as mean ± SD. *, **, ***and **** represent *p* < 0.05,0.01, 0.001and 0.0001, respectively, compared to the control group. The symbols # and ### represent *p* < 0.05 and 0.001, respectively, for comparisons between MSC group and hydrogel group. The symbols ^, ^^, ^^^and ^^^^ represent *p* < 0.05,0.01, 0.001and 0.0001 for comparisons between MSC-laden hydrogel and hydrogel only group. The symbols && and &&&& represent *p* < 0.01 and 0,0001 for comparisons between MSC-laden hydrogel and MSC group. Statistical significance is determined using Tukey's post-hoc test following a one-way ANOVA (p < 0.05); **C** Mean area-under-curve (AUC) of individual wounds of each group, n = 5 for all groups. The AUC of individual wounds in the treatment groups was normalized against the mean AUC of vehicle controls within the same experimental runs. All data are presented as mean ± SD. **, and **** represent *p* < 0.01, and 0.0001, respectively, by Tukey’s *post-hoc* test when statistical significance by One-way ANOVA (*p* < 0.05) is obtained; **D** Representative hematoxylin and eosin (H&E) of wounds on day 7 and day 11 for each group, scale bar = 500 μm and 100 μm. Quantification of granulation tissue gap and epidermal thickness on day 11 is shown on the right, n = 5 for all groups. All data are presented as mean ± SD. *, **, ***and **** represent *p* < 0.05,0.01, 0.001and 0.0001, respectively, by Tukey’s *post-hoc* test when statistical significance by One-way ANOVA (*p* < 0.05) is obtained; **E** Masson’s trichrome staining (MTS) of wounds on day 7 and day 11 for each group, scale bar = 500 μm and 100 μm. Quantification of collagen deposition of the wounds on day 10, n = 5 for all groups. All data are presented as mean ± SD. * and **** represent *p* < 0.05, and 0.0001, respectively, by Tukey’s *post-hoc* test when statistical significance by One-way ANOVA(*p* < 0.05) is obtained

**Fig. 9:**
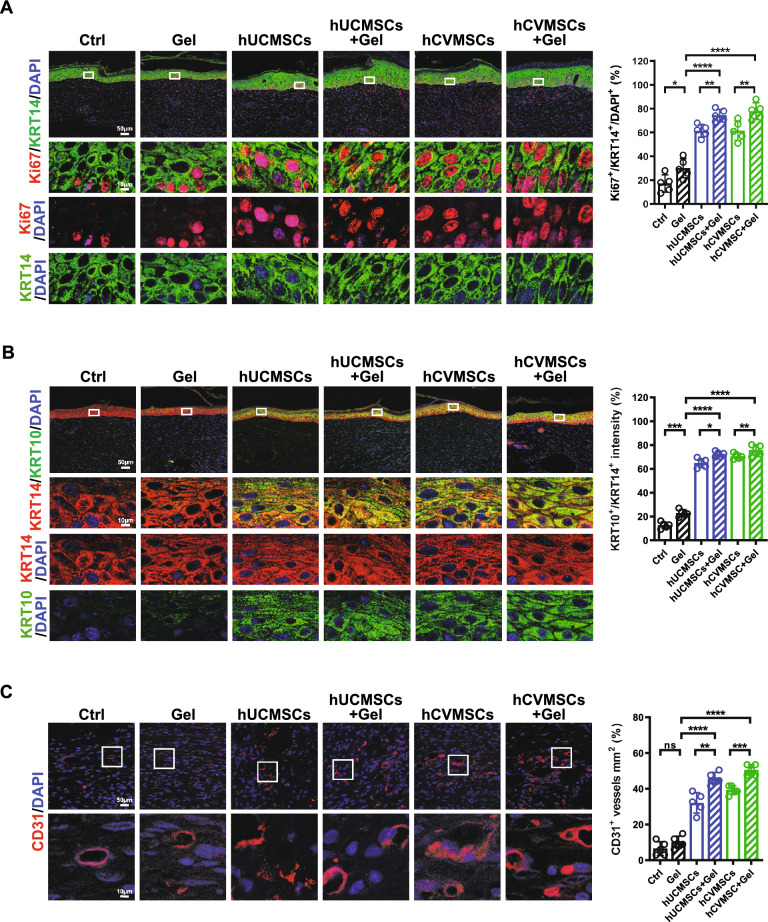
PEGDA/SA/Col-I hydrogel encapsulation enhances MSC therapeutic efficacy for diabetic wound healing. **A** Representative immunofluorescence (IF) images of Ki67 and KRT14 staining in wounds on day 11 for each experimental group. Quantification of Ki67 + and KRT14 + double positive cells is shown on the right; **B** Representative IF images of KRT10 and KRT14 staining in wounds on day 11 for each experimental group. Quantification of the average intensity percentage of KRT10 + versus KRT14 + is shown on the right; **C** Representative IF images of CD31 staining in wounds on day 11 for each experimental group. Quantification of CD31 + cells per unit area is shown on the right. All data are presented as mean ± SD, n = 5 for all groups. Scale bar = 50 μm and 10 μm. *, **, *** and **** represent *p* < 0.05, 0.01,0.001 and 0.0001, respectively, by Tukey’s *posthoc* test when statistical significance by One-way ANOVA(*p* < 0.05) is obtained

We then proceeded to evaluate the therapeutic effects of the MSC-laden PEGDA/SA/Co-I hydrogel on promoting diabetic wound healing in vivo. Compared to the vehicle and gel-treated groups, wounds treated with either hUCMSCs or hCVMSCs showed a consistent and accelerated reduction in wound size (Fig. 8A, B). Notably, both hUCMSC-laden hydrogel and hCVMSC-laden hydrogel demonstrated a more significant acceleration in the healing process. On Day 9, the average relative wound sizes were as follows: 24.7 ± 9.4% (vehicle), 18.5 ± 11.3% (hydrogel), 9.7 ± 7.3% (hUCMSCs), 5.4 ± 3.6% (hUCMSC-laden hydrogel), 13.0 ± 8.3% (hCVMSC), and 5.3 ± 4.7% (hCVMSC-laden hydrogel). By Day 11, both MSC-treated groups and MSC-landen hydeogel groups showed nearly complete closure, while the average wound sizes of the control group and hydrogel-only group were 11.6 ± 4.0% and 5.3 ± 3.6% (Fig. 8B). This was also reflected in the smaller wound healing areas under the curve (AUC) in the hUCMSC-laden hydrogel and hCVMSC-laden hydrogel groups (Fig. 8C). Additionally, wounds treated with hMSC-laden hydrogels exhibited decreased granulation tissue gaps and increased epidermal thickness compared to those treated with hydrogel-only or cell-only, indicating enhanced re-epithelialization and improved healing (Fig. 8D). MST staining revealed a higher density and intensity of collagen fibers in the dermal connective tissue of the MSC-laden hydrogel groups compared to the cell-treated and gel-treated groups (Fig. 8E). Furthermore, we observed a significant increase in proliferating KRT14^+^ cells (KRT14^+^/Ki67^+^) in the MSC-laden hydrogel groups compared to the gel-only and cell-only groups (Fig. 9A). The ratios of KRT10 to KRT14 were also significantly higher in the MSC-laden hydrogel groups (Fig. 9B). Finally, enhanced vascularization was evident in the wounds treated with hUCMSC-laden hydrogel and hCVMSC-laden hydrogel, as demonstrated by the higher density of CD31^+^ vessels compared to the cell-only and gel-only groups. Collectively, our results demonstrate that the PEGDA/SA/Col-I hydrogel significantly enhances the therapeutic effects of hUCMSCs and hCVMSCs on diabetic wound healing.

## Discussion

DFUs present a significant clinical challenge due to their multifactorial pathogenesis and poor healing outcomes with conventional care. MSC-based therapies have emerged as a promising alternative, demonstrating potential to modulate DFU pathology through various preclinical and clinical investigations. While diverse MSC sources have been explored, consensus is lacking on optimal donor specifications. In this study, we utilized three distinct types of MSCs: hUC-MSCs, hCV-MSCs, and hDC-MSCs. hUC-MSCs are the most widely used perinatal tissue-derived MSCs, while hCV-MSCs and hDC-MSCs represent MSCs sourced from the fetal and maternal sides of the placenta, respectively. These MSC types were chosen for comparison due to their distinct origins. The selection of these MSC types allows for a comprehensive comparison of their unique characteristics, including their secretory profiles and potential therapeutic applications. Our results demonstrated that hUCMSCs and hCVMSCs exhibited superior wound healing properties compared to hDCMSCs. Protein analysis identified both common and distinct secretory factors among these MSC types. Furthermore, we found that MSCs derived from perinatal tissues promote cell proliferation and migration in keratinocytes and endothelial cells, with the PI3K/AKT signaling pathway serving as a common regulatory mechanism. The optimized PEGDA/SA/Col-I hydrogel provided a protective and supportive environment, further amplifying the therapeutic effects of MSCs in wound healing.

In the field of regenerative medicine, placenta-derived MSCs (PD-MSCs) have emerged as a promising therapeutic option for diabetic wound healing. PD-MSCs can be sourced from various tissues within the placenta, including the amniotic fluid, amniotic membrane, chorionic plate, chorionic villi, decidua basalis, and the complete placenta. Despite potential variations in their properties based on their specific origin, numerous studies have indicated that PD-MSCs exhibit remarkable differentiation potential, self-renewal abilities, and low immunogenicity, making them suitable for promoting tissue repair [37]. Notably, MSCs derived from fetal placental tissues exhibit significantly higher proliferative capacity compared to those from maternal placental tissues [38]. Understanding these nuanced differences is crucial for optimizing their application in diabetic wound healing. In this study, we conducted a comparative analysis by evaluating the therapeutic potential of hCVMSCs and hDCMSCs, in a diabetic wound healing model using db/db mice. Our findings revealed that while both hCVMSC- and hDCMSC-treated groups exhibited accelerated wound healing compared to the vehicle group, hCVMSCs demonstrated superior wound-healing kinetics, comparable to the extent observed with hUCMSCs (Fig. 1B–D). IF analysis further corroborated these findings, showing a significant increase in KRT14^+^/Ki67^+^ cells and an increased ratio of KRT10/KRT14 in the hCVMSCs group compared to the hDCMSCs group, indicating enhanced keratinocyte proliferation and maturation (Fig. 2A, B). Additionally, hCVMSCs exhibited a greater effect on promoting angiogenesis, as evidenced by an increased number of CD31^+^ microvessels compared to the hDCMSC-treated group. These data suggest that hCVMSCs derived from fetal tissues are more effective in promoting critical steps in wound healing compared to hDCMSCs derived from maternal tissue. Notably, our in vitro functional studies demonstrated similar promoting effects of hCVMSCs and hDCMSCs on cell proliferation, migration and tube formation, possibly due to the lack of injury in the culture system, which cannot fully recapitulate the hostile healing environment in the context of diabetes. hUC-MSCs have been extensively studied for their potential in treating DFUs. Numerous studies have demonstrated that hUCMSCs exhibit strong paracrine effects, secreting a variety of growth factors and cytokines that promote cell proliferation, enhance angiogenesis, reduce inflammation, and accelerate skin tissue regeneration [39–41]. Our study has demonstrated consistent results, highlighting its superior repair effectiveness on diabetic wound healing. While autologous stem cell transplantation using adult MSCs has clear advantages, it may not be suitable for patients experiencing advanced aging or specific disease conditions, making perinatal MSCs a viable alternative.

Our molecular comparison of the secretome profiles of hUCMSCs, hCVMSCs, and hDCMSCs has provided valuable insights into their common and distinct biological pathways associated with wound healing. The identification of 435 common proteins, primarily involved in ECM organization and wound healing, underscores the fundamental role of ECM regulatory proteins in MSC-mediated tissue repair. Gene ontology (GO) analysis highlighted these proteins' involvement in critical functions such as blood coagulation, keratinocyte or endothelial cell migration, differentiation and proliferation, and angiogenesis (Fig. 4E). The enrichment of proteins like THBS1, SERPINE1, ANXA1, LOX, and ITGB1 in all three MSC types further emphasizes their pivotal roles in the wound healing process. For instance, THBS1 has been shown to modulate cell-to-cell and cell-to-matrix interactions, facilitating tissue repair and angiogenesis [42, 43]. SERPINE1 plays a crucial role in regulating fibrinolysis and stabilizing the extracellular matrix, which is vital for proper wound closure [44, 45]. ANXA1 has anti-inflammatory properties and promotes the resolution of inflammation, thereby aiding in the healing process [46, 47]. LOX is involved in the cross-linking of collagen and elastin, which is essential for the structural integrity and strength of the healed tissue [48, 49]. ITGB1 is a key mediator of cell adhesion and migration processes that are fundamental to the re-epithelialization of wounds [50, 51]. Notably, while hCVMSCs and hUCMSCs displayed greater similarity in their secretome (Fig. S4B) which underscores their comparable efficacy in promoting wound healing, our data also revealed significant differences in the secretory factors among the three MSC types. For instance, numerous ribosomal proteins, including RPL10A, RPL4, RPS13, RPS18 and elongation factors EIF2S3, EIF3A, EIF3D and ETF1 are uniquely enriched in hUCMSCs, suggesting that hUCMSCs have the ability to modulate protein synthesis and proteome homeostasis (Fig. S5B). The distinct secretory factors unique to each MSC type highlight the importance of tailoring MSC selection to specific clinical needs. For instance, the enrichment of translation regulation and amino acid metabolic process-related proteins in hUCMSCs suggests a potential advantage in scenarios requiring rapid protein synthesis and cellular metabolism. Conversely, the ECM organization and immune response proteins in hCVMSCs could be pivotal for inflammatory modulation and matrix remodeling, while the blood coagulation and tissue homeostasis proteins in hDCMSCs may confer superior efficacy in hemostatic applications. Our findings align with previous studies demonstrating that MSCs from different tissue sources possess distinct secretory profiles, leading to varied therapeutic outcomes [52–54]. This reinforces the notion that the ideal MSC source must be carefully selected based on the specific therapeutic context.

The PI3K/AKT signaling pathway plays a crucial role in various cellular processes essential for wound healing, including cell proliferation, migration, and angiogenesis. Activation of this pathway has been consistently shown to enhance these processes, thereby promoting effective wound repair [55]. Indeed, both animal and clinical trials on diabetes have confirmed that activation of the AKT pathway participates in topical insulin-induced wound healing [56]. In our study, we observed that the secretome from three types of perinatal MSCs significantly activated the PI3K/AKT pathway in HaCaT and HUVEC cells, whereas suppression of this pathway by either PI3K or AKT inhibitor abolished the promoting effects of MSCs on cell proliferation and migration (Figs. 5, 6). Our findings align with previous reports demonstrating the importance of the PI3K/AKT pathway in MSC-mediated wound healing [57]. For instance, a study by Li et al. demonstrated that human amniotic mesenchymal stem cell (hAMSC) transplantation in a mouse model of second-degree burn injury enhanced proliferation, inhibited apoptosis of skin cells, and promoted angiogenesis through the PI3K/AKT signaling pathway [58]. Additionally, a recent study revealed that exosomes derived from BMMSCs facilitated diabetic wound healing by restoring keratinocyte autophagy [59]. These studies, along with ours, provide strong evidence that the PI3K/AKT pathway is one of the major and common signaling pathways underlying the therapeutic effects of different types of MSCs. In an effort to identify the potential components within the MSC secretome responsible for PI3K/AKT pathway activation, we noted the presence of various ECM proteins known to interact with integrin receptors on the cell surface. ECM proteins such as collagen VI, collagen IV, FN1 and Laminin are highly enriched in the secretome derived from the three types of MSCs (Fig. 4G). These proteins can bind to αβ1 integrins. Integrins are transmembrane receptors that, upon activation, induce changes in intracellular signaling pathways, notably the PI3K/AKT pathway. Besides, integrin β1 (ITGB1) is the principal receptor that mediates the connection between epidermal stem cells and extracellular matrix. Upon ligand binding, ITGB1 accelerates the proliferation of epidermal stem cells via activating the downstream signaling pathways. Given that ITGB1 is also highly enriched in our secretome, we speculate that this integrin-ECM interaction could be a critical mechanism underlying the activation of PI3K/AKT pathway, offering valuable insights into the mechanisms driving the therapeutic effects of perinatal MSCs in wound repair and regeneration.

The encapsulation of MSCs within hydrogels presents a promising strategy for enhancing stem cell-based therapies, particularly in the challenging context of diabetic wound healing. Our study aimed to improve upon existing hydrogel formulations by incorporating PEGDA and collagen I into an SA hydrogel matrix. The resultant PEGDA/SA/Col-I hydrogel demonstrated a unique balance of mechanical and biological properties that collectively enhance MSC viability and therapeutic efficacy. One of the critical findings was the optimal compressive modulus of the PEGDA/SA/Col-I hydrogel. While a higher compressive modulus, such as that of the 12% PEGDA hydrogel, provides substantial structural integrity, it compromises flexibility and conformability to irregular wound surfaces. In contrast, the PEGDA/SA/Col-I hydrogel, with its intermediate compressive modulus, offers sufficient structural support while maintaining the necessary flexibility. This characteristic is particularly important for wound dressings that must adapt to the dynamic movements of the skin without fracturing. Additionally, the PEGDA/SA/Col-I hydrogel boasts several other advantages. Its biocompatibility ensures minimal adverse reactions, promoting a favorable healing environment. The sodium alginate component enhances hydration retention, creating a moist milieu that supports cell migration and proliferation. Moreover, the collagen type I component mimics the natural ECM, facilitating enhanced cell adhesion and promoting regenerative activities. Our in vitro experiments confirmed that the PEGDA/SA/Col-I hydrogel not only provided a protective environment against oxidative stress but also promoted continued growth and protein secretion of MSCs (Fig. 7B–F). These findings suggest that the PEGDA/SA/Col-I hydrogel can create a more conducive microenvironment for MSCs, enhancing their therapeutic potential. In consistent with the in vitro experiments, the MSC-laden PEGDA/SA/Col-I hydrogel markedly accelerated diabetic wound healing, as evidenced by faster wound closure, enhanced re-epithelialization, and increased collagen deposition (Fig. 8). Notably, the observed increase in vascularization and enhancement of epidermal proliferation and maturation underscores hydrogel’s ability to promote essential wound healing processes (Fig. 9). When considering other biomaterials for MSC encapsulation in wound healing, several alternatives have been evaluated. For instance, gelatin-based hydrogels have shown promise due to their biocompatibility and biodegradability, facilitating cell adhesion and growth [60]. Similarly, hyaluronic acid hydrogels have been recognized for their ability to retain moisture and promote cellular migration, further enhancing healing outcomes [61, 62]. Additionally, fibrin-based scaffolds have been utilized to support cell survival and proliferation, leveraging their natural role in wound healing [63, 64]. Collectively, the PEGDA/SA/Col-I hydrogel represents a significant advancement in the design of hydrogel-based wound dressings, offering a suitable scaffold for skin repair while protecting MSCs and enhancing their regenerative functions. Future comparative studies that evaluate these various biomaterials in similar in vivo models will provide deeper insights into their relative efficacies and mechanisms of action, ultimately guiding the optimization of MSC therapies for wound healing.

## Conclusion

Our study demonstrates that MSCs derived from perinatal tissues, particularly hUCMSCs and hCVMSCs, exhibit superior therapeutic potential for diabetic wound healing compared to hDCMSCs. The optimized PEGDA/SA/Col-I hydrogel further enhances the regenerative functions of MSCs by providing a supportive and protective environment. These findings underscore the importance of selecting the appropriate MSC source and hydrogel formulation to maximize therapeutic efficacy in DFU treatment. Future research should aim to further elucidate the molecular mechanisms underlying MSC-mediated wound healing and explore the clinical translation of these promising therapeutic strategies.

### Limitation of the study

While our study offers significant insights into the therapeutic potential of MSCs derived from perinatal tissue for diabetic wound healing, there are areas that warrant further exploration. Firstly, although our mass spectrometry data indicated the presence of immune regulatory factors in the MSC secretome, we did not specifically investigate immunoregulation in our studies. Understanding this aspect could provide a more comprehensive picture of the MSCs' therapeutic mechanisms. Secondly, while our in vitro studies showed that the MSC-derived secretome enhances keratinocyte and endothelial cell functions, we did not assess the effects of the secretome in vivo. This presents an opportunity for future research, especially considering the PEGDA hydrogel's known efficacy in releasing bioactive factors for tissue repair. Addressing these areas in subsequent studies could further validate and extend the promising findings of our current work.

## Supplementary Information

Additional file1 (PDF 19843 kb)

## Data Availability

The datasets used during the current study are available from the corresponding author on reasonable request and the proteomics data has been deposited in MassIVE 10.25345/C5F47H56X.

## Abbreviations

DFUs: Diabetic foot ulcers
MSCs: Mesenchymal stem cells
hUCMSCs: Human umbilical cord MSCs
hCVMSCs: Human chorionic villi MSCs
hDCMSCs: Human decidua basalis MSCs
HUVECs: Human umbilical vein endothelial cells
MEFs: Mouse embryonic fibroblasts
PEGDA: Polyethylene glycol diacrylate
SA: Sodium alginate
Col-I: Collagen I
THBS1: Thrombospondin 1
SERPINE1: Serpin family E member 1
ANXA1: Annexin A1
LOX: Lysyl oxidase
ITGB1: Integrin beta-1
ECM: Extracellular matrix
CM: Conditional media
PBS: Phosphate-buffered saline
PVDF: Polyvinylidene difluoride
TBST: Tris-buffered saline containing 0.1% Tween-20
OGD/R: Oxygen-glucose deprivation/reoxygenation
ROI: Region of interest
ANOVA: One-way analysis of variance
MST: Masson's trichrome
KRT14: Keratin 14
KRT10: Keratin 10
PCA: Principal component analysis
GO: Gene ontology
SPP1: Secreted phosphoprotein 1
HSP90B1: Heat shock protein 90 beta family member 1
VEGFC: Vascular endothelial growth factor C
IGF2: Insulin-like growth factor 2
PI3K: Phosphoinositide 3-kinase
