# Supplementary material for: Mesenchymal stem cells from perinatal tissues promote diabetic wound healing via PI3K/AKT activation
Source: Stem Cell Res Ther. 2025 Feb 8;16:59. doi: 10.1186/s13287-025-04141-8 (PMC11807333; doi:10.1186/s13287-025-04141-8)
Supplement: Supplementary file 1 — Additional file1 (PDF 19843 kb) [file 13287_2025_4141_MOESM1_ESM.pdf]

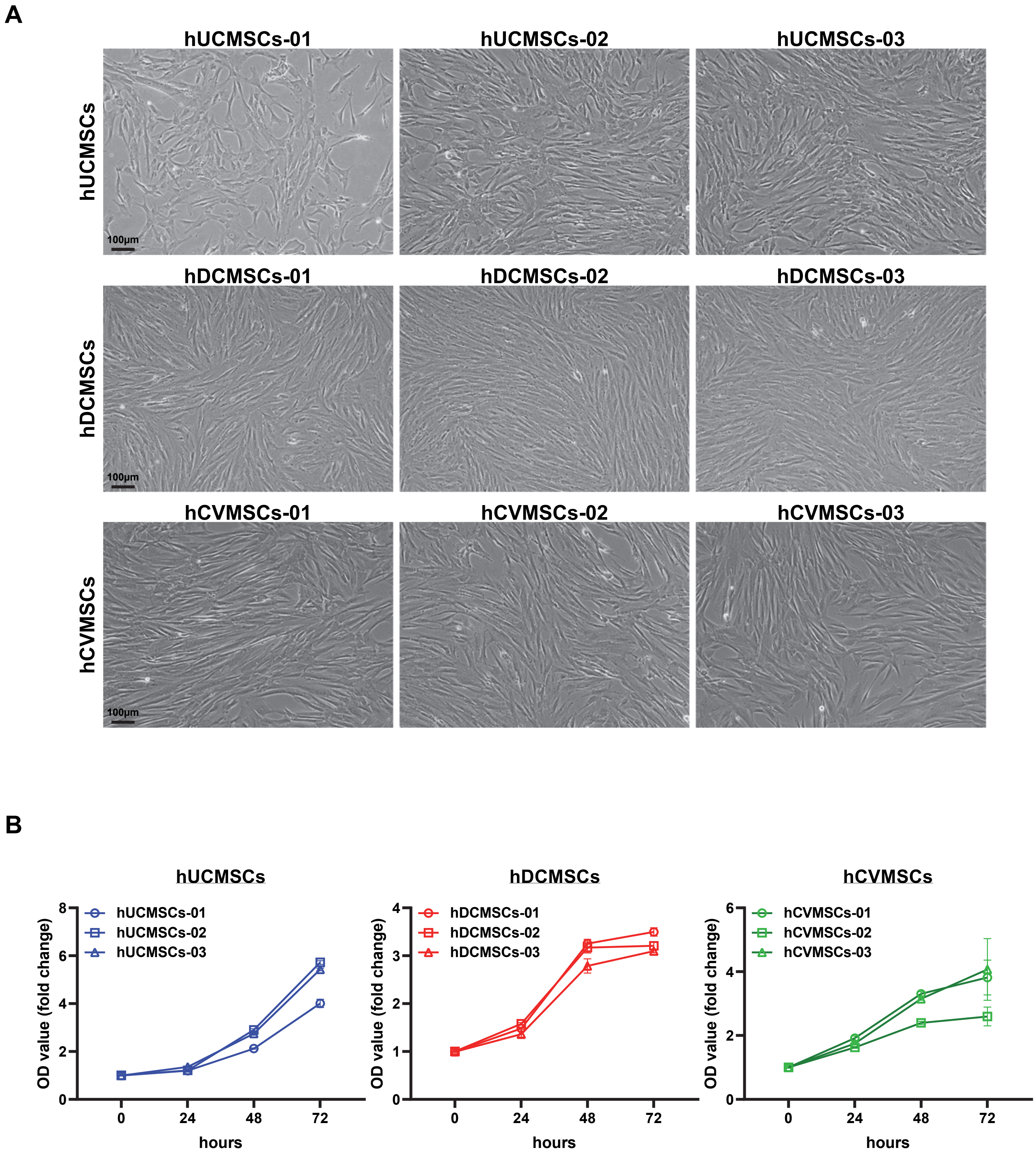

A
hUCMSCs-01
hUCMSCs-02
hUCMSCs-03
hUCMSCs
100μm
hDCMSCs-01
hDCMSCs-02
hDCMSCs-03
hDCMSCs
100μm
hCVMSCs-01
hCVMSCs-02
hCVMSCs-03
hCVMSCs
100μm
B
hUCMSCs
hUCMSCs-01
hUCMSCs-02
hUCMSCs-03
OD value (fold change)
0
2
4
6
8
0
24
48
72
hours
hDCMSCs
hDCMSCs-01
hDCMSCs-02
hDCMSCs-03
OD value (fold change)
0
1
2
3
4
0
24
48
72
hours
hCVMSCs
hCVMSCs-01
hCVMSCs-02
hCVMSCs-03
OD value (fold change)
0
2
4
6
0
24
48
72
hours

Figure S1. Morphology of perinatal MSCs. A) Phase-contrast images of hUCMSCs, hDCMSCs and hCVMSCs derived from 3 donors (scale bar: 100 μm); **B)** Cell proliferation of perinatal MSCs was assessed by MTT assay. Experiments were repeated at least three times.

A

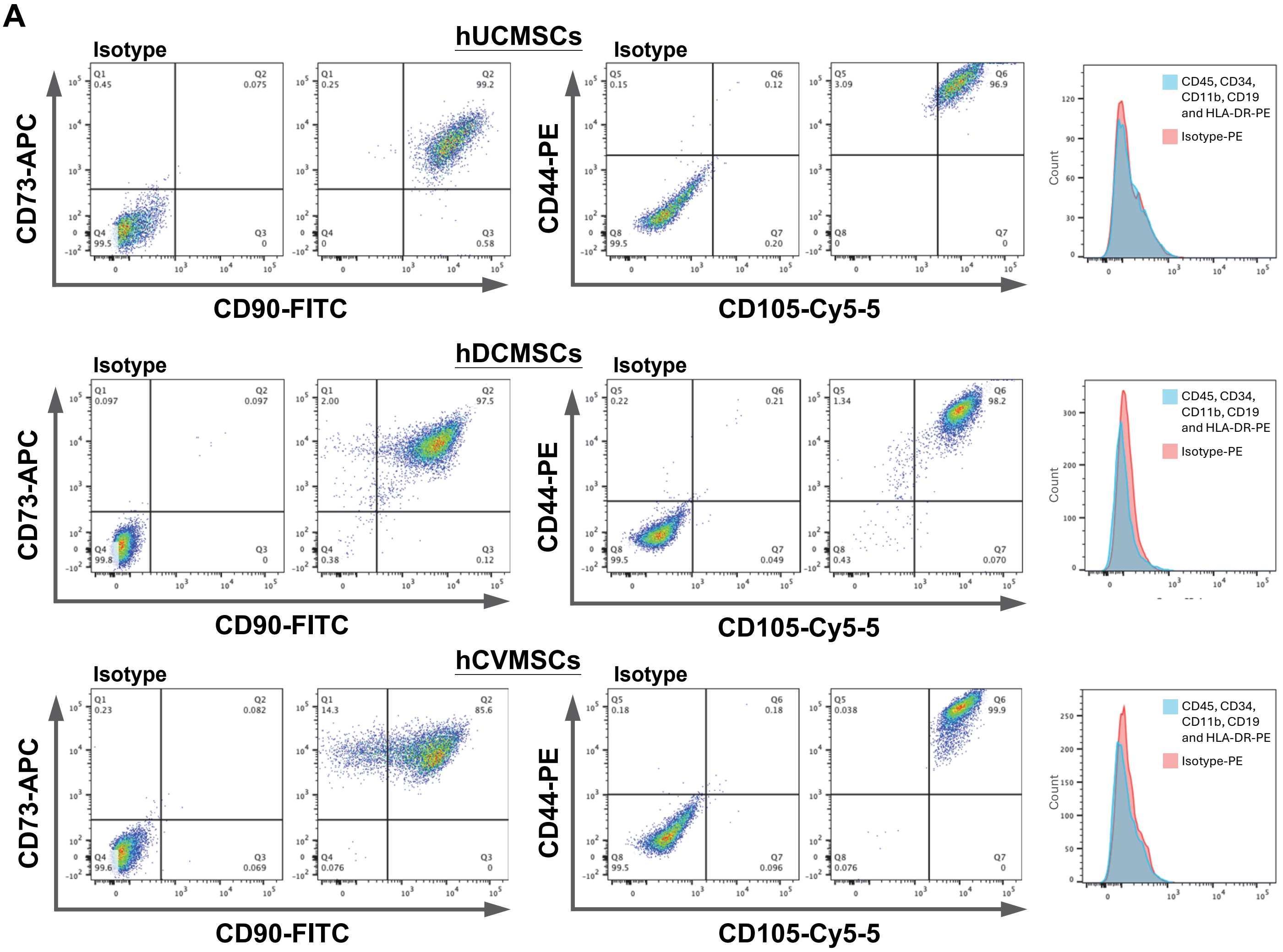
hUCMSCs
hDCMSCs
hCVMSCs
Isotype
CD73-APC
CD90-FITC
CD44-PE
CD105-Cy5-5
Count
CD45, CD34, CD11b, CD19 and HLA-DR-PE
Isotype-PE

B

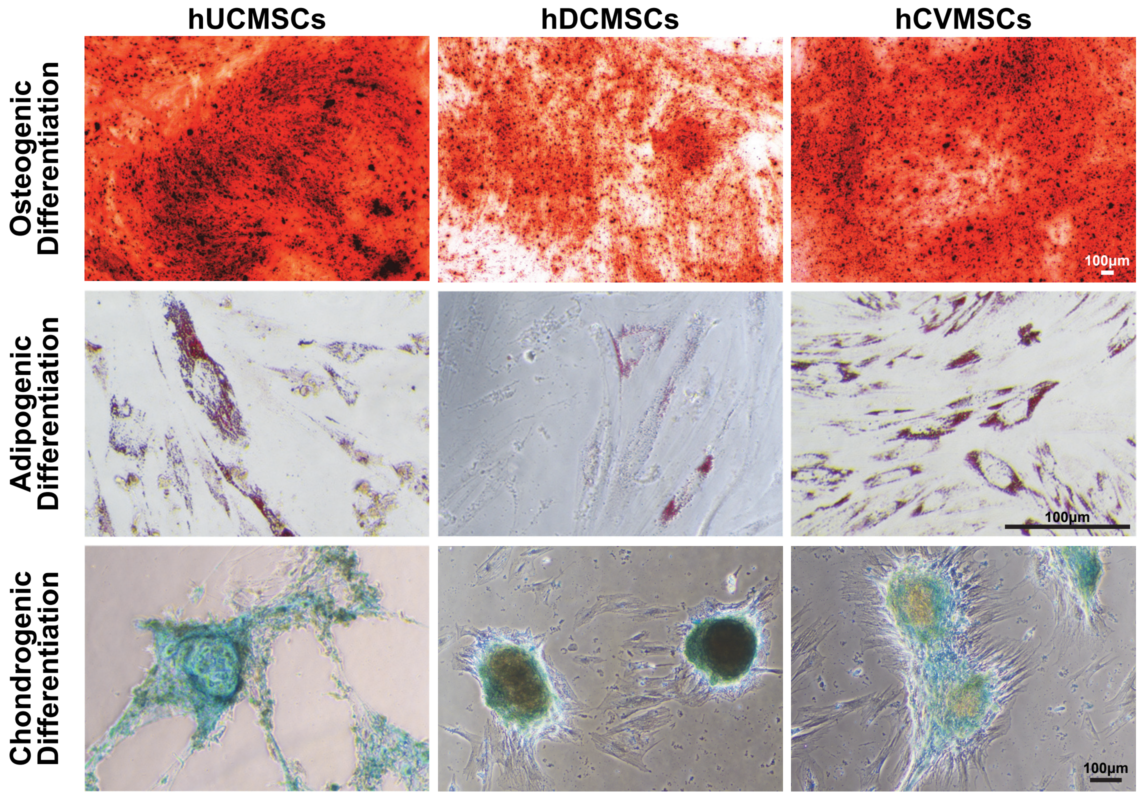
hUCMSCs
hDCMSCs
hCVMSCs
Osteogenic Differentiation
Adipogenic Differentiation
Chondrogenic Differentiation
100μm
100μm
100μm

Figure S2. Characterization of perinatal MSCs. A) Flow cytometry analysis of perinatal MSCs using positive markers, CD90, CD105, CD44 and CD73 and negative markers, CD34, CD11, CD19, CD45 and HLA-DR; **B)** Perinatal MSCs could undergo osteogenic, adipogenic and chondrogenic differentiation (scale bar = 100 μm) as demonstrated by Alizarin red, Oil red O, and Alcian blue staining. Experiments were repeated at least three times.

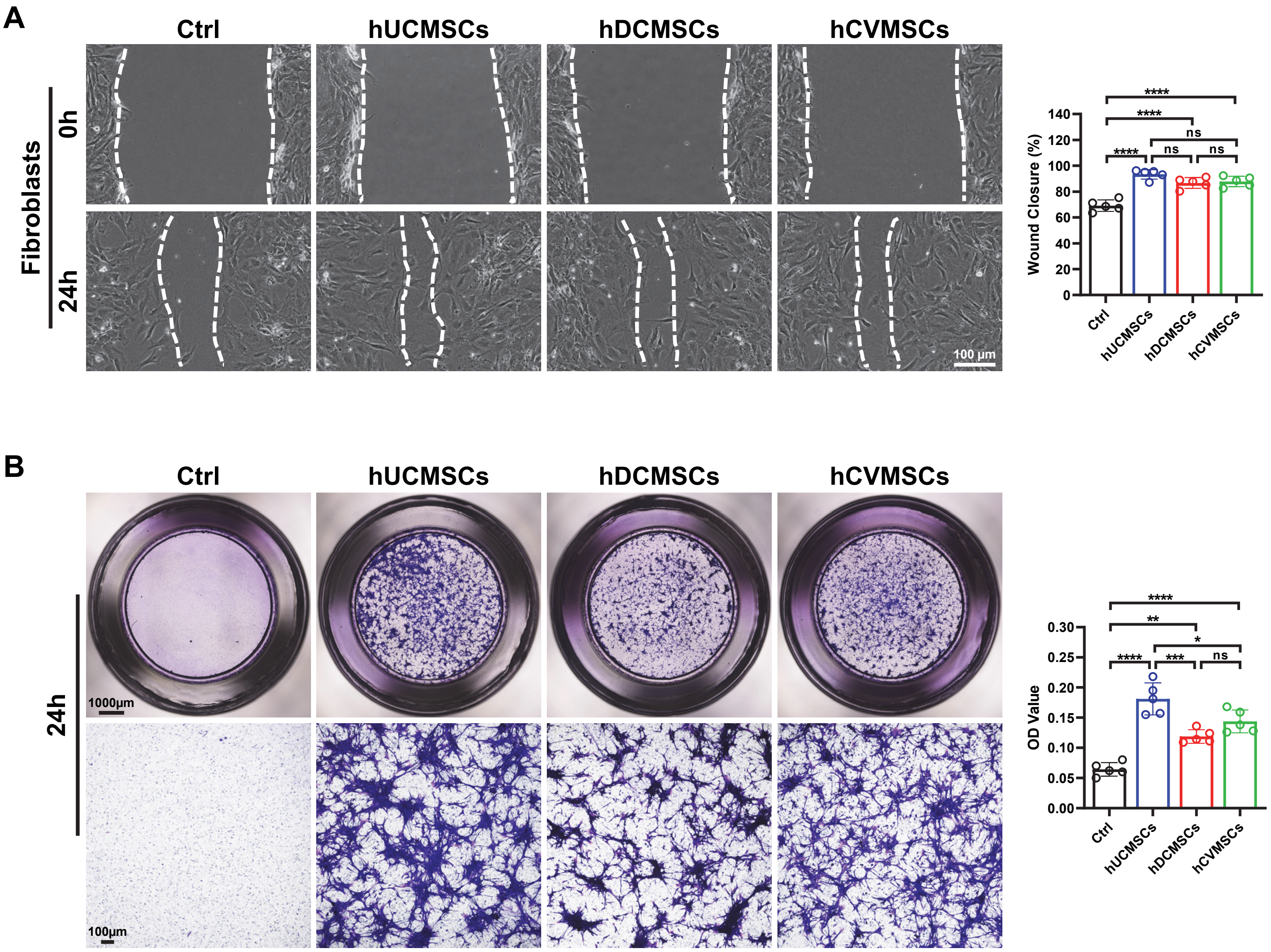
A
Ctrl
hUCMSCs
hDCMSCs
hCVMSCs
Fibroblasts
0h
24h
100 μm
Wound Closure (%)
140
120
100
80
60
40
20
0
****
****
ns
****
ns
ns
Ctrl
hUCMSCs
hDCMSCs
hCVMSCs
B
Ctrl
hUCMSCs
hDCMSCs
hCVMSCs
24h
1000μm
100μm
OD Value
0.30
0.25
0.20
0.15
0.10
0.05
0.00
****
**
*
****
***
ns
Ctrl
hUCMSCs
hDCMSCs
hCVMSCs

Figure S3. The secretome derived from perinatal MSCs promotes MEF migration and hBMMSC recruitment *in vitro*. A) Images from the wound scratch healing assay for mouse embryonic fibroblasts (MEF) treated with different secretome, scale bar = 100 μm. Quantification of MEF migration is shown on the right. Experiments were repeated at least three times; **B)** Transwell migration assay was used to analyze the recruitment of hBMMSCs by the secretome derived from perinatal MSCs. Scale bar =1000 μm and 100 μm. Quantification of DMSO-dissolved crystal violet at OD (490 nm) is shown on the right. Experiments were repeated at least three times. All data are presented as mean ± SD. *, **, ***, and **** represent $p < 0.05$, 0.01, 0.001 and 0.0001, respectively, by Tukey's *post-hoc* test when statistical significance by One-way ANOVA ($p<0.05$) is obtained.

A

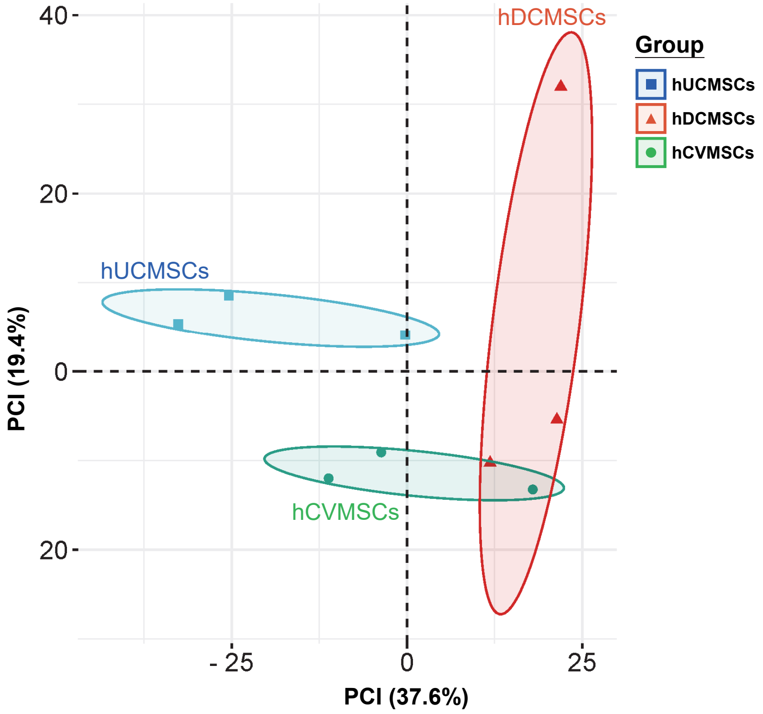

B

hUCMSCs						hDCMSCs	hCVMSCs		hUCMSCs & hDCMSCs	hUCMSCs & hCVMSCs						hDCMSCs & hCVMSCs
FASN	MAP1LC3B2	TAX1BP3	ATIC	DCBLD1	CRYZ	TUBA1A	C4B_2	CXCL5	SET	HMCN1	TINAGL1	RANBP1	SKP1	PGM1	DIRAS2	TNXB
TUBB2A	ACOT7	XPO1	GBP1	SCFD1	MGAT5	TUBA4A	TUBB4B	ECE1	MFAP2	SPTAN1	CPXM1	TFPI2	RPL19	NNMT	CENPE	SERPING1
EEF1A1	DDX3X	EIF3D	SHMT2	DNAJC9	SCRN1	KRT6B	TUBA3C	MCAM	CNDP2	PLEC	PFKP	PSMB2	TWF2	PRCP	EIF4G1	EEF1A1P5
TUBB3	CAPNS1	EIF3H	RPL4	ARAP2	AIMP1	KRT2	PSG4	PPIC	DYNC1H1	CSPG4	UGP2	USP14	MOXD1	NAMPT	RPL18	MASP1
LAMC2	FH	PSMD3	DLST	LXN	CIRBP	KRT14	CFB	SUB1	RPS2	KRT8	PSMA7	ARPC4	SERBP1	USP5	GALNT1	ALDH1A1
TUBA1C	FDPS	ZNF207	CAPG	COPS4	DCTN1	KRT16	PSG5	UFM1	KRT9	CPA4	UGDH	PPP2CB	CPPED1	RARRES1	EIF3I	MMP3
MAP1B	DARS1	GET3	MATR3	ANP32E	DSC3	COMP	STAT1	RPL8	CDH6	SPTBN1	PGLS	CCT2	NUCKS1	HMGA2	DYNC1I2	THBS3
TUBB6	ACO1	AP1G1	RPS9	PBDC1	PTPA	ENO3	GAS1	MRPS35	PDAP1	COL11A1	GSR	CAVIN1	ACBD3	NAGLU	ADAM15	APLP2
MYH10	GART	HSBP1	QARS1	ULBP2	PCBP2	EIF5AL1	CFD	DNAH14	VNN2	CCT8	KRT19	ST13P4	GLOD4	MANF	PICALM	CCN1
HNRNPR	PAICS	SNRNP200	LIMS1	API5	PPP1R7	TUBB1	CXCL10	FRMPD1	H1-5	TUBA1B	HMGB1	RTN4	HEBP1	RPS20	ASAH1	F2
HSPA2	MAPK1	EIF3J	GSS	TAOK3	TSN	F10	ANGPTL2	LIN54	CANX	MANBA	ANPEP	STRAP	SEPTIN9	COPS2	FHL1	HLA-B
HNRNPA1	KIF5B	SEC24D	LMAN1	GREM2	TRIP13	APOE	CFL2	PLXDC2	RPL3	HAPLN1	CPE	RUVBL1	GPC6	RPS3A	CTTN	MMP11
CBR1	ACTR1B	IPO7	CENPF	MYG1	DDX1	C7	GREM1	ABI3BP	HSPE1	GDI1	PSMA2	VPS35	NIBAN2	PSMC6	LASP1	SULF1
APEX1	TMPO	OXSR1	SRP9	ZSWIM6	GCN1	STMN1	CBR3	RBM45	RPS21	TCP1	PSMA4	GBE1	GLO1	DYNLL1	PSMD6	ANGPT1
RDX	ME1	BAG2	SERPINB8	NRXN3	TFG	YBX1	SETSIP	SCUBE3	LTBP4	CCT3	TARS1	CKAP4	PSME1	PAFAH1B2	PDGFRL	REN
FLT1	RAB5C	DDAH2	METAP2	NANS	GLG1	PSMA8	ISG15	EMILIN2	ABCA10	DPYSL2	ANP32A	DSG2	ARHGAP1	GSTO1	MAN2A1	PTN
IARS1	AK2	STAG3L1	BCAM	EXTL2	TBCB	HMGB1P1	SOD3	ANTXR1	PDLIM5	NRP2	HDGF	MVP	PSMD2	CXCL6	QPCT	CHI3L1
MYH14	EIF3B	PLAU	RPL14	EPHA6	CNN2	DNASE2	CXCL2	IQCG	MAP2K1	PGD	SEC13	PCBP1	G3BP1	RPL24	FAM3C	RARRES2
ADAMTSL1	UBE2K	TFRC	ALDH5A1	LAMTOR3	S100A13	PEX7	MX1	SIL1		NPEPPS	EIF6	ELOC	DCTN2	TNFAIP6		CANT1
HSPA1A	ARF3	GBA	SMS	ENOPH1	SH3GL1	LECT2	CRABP2	TIGAR		DPYSL3	LRRC17	SEPTIN7	RSU1	RBM3		PCDH18
GNAI3	RPS18	ITGB2	LIMK2	NAGK		DENR	BTD	CYRIB		EXT1	RRBP1	TNFRSF11B	UBE2V2	CYCS		HNRNPA1L2
PCNA	ETF1	SERPINB2	SEC24C	TRHDE		MPDZ	SFRP1	ETAA1		SLC3A2	PA2G4	PDXK	ZYX	AK9		COL14A1
NME1	RPL10A	SERPINA5	ADAR	CDV3		VNN1	FABP5P3	OR5AC2		HSPA9	GALNT10	COPE	PARK7	LPCAT2		CA12
AARS1	RAC1	ICAM1	ITGA1	MACF1		AMY1B	PDLIM1	CGN		EIF4A1	CAND1	B4GALT4	EIF3CL	VN1R5		F9
COPA	AP2B1	GLA	EIF3E	CNPY2		C5	MYO1C	COPG2		EIF5A	OTUB1	IDH1	TOR1B	BBS7		APOA1
CSE1L	TXNDC17	RHOC	PSMC1	PPP2R2C		FGB	GPC4	CTSF		PSAT1	FHL2	PNP	ARPC5	CHST14		C4BPA
TGFB2	ACAT2	MME	PSMC5	SUGT1		FGG	CREG1	DNAJB4		SEC23A	PPA1	RBP4	TXNL1	PLBD2		CFI
XYLT1	RAB1B	CD63	RPS13	RTCB		LCAT	RASSF9	DPP7		CCT7	PTPRK	ITGAV	SEC22B	DYNLRB2		DBI
YWHAH	PCDH10	DLD	SNRPE	RBM8A		CD55	SEC31A	ADAMTS7		CCT5	LGMN	CSF1	ATP6AP2	CEMIP		ANXA4
DHX9	NTM	PTPRF	TMSB4X	LIPG		F5	SBF1	EPDR1		SND1	ARPC3	LTA4H	AP2A2	STON2		SRGN
PRMT1	PRPF19	ESD	CNBP	HEBP2		MDK	CCDC175	YBX2		GALNT2	NARS1	CTSA	CSTB	PDCD1LG2		IGF2R
HNRNPDL	HYOU1	SLC2A1	RPL23	BZW2		GRIA1	FUCA1	TRPC6		ILF2	CLN5	CCL2	SHBG	CORO1B		CKB
PFDN6	AMIGO2	P4HA1	RPS24	CABIN1		RBMXL3	RPN1	PDLIM3		DDX39B	FSTL3	SRM	CAPN1	GINS3		ARF4
PHGDH	FABP5	SNRPB	PPP3R1	RUVBL2		ANKS4B	C2	RNF180		VAT1	HLA-H	LMNB1	ITGA5	ESYT1		LAMA1
SERPINB7	KHDRBS1	JUP	PSPH	HSPB11		C9orf43	PDGFRB	WDR86		PGM3	THY1	PSMA1	CXCL8	AP1M1		ADM
GLRX3	RBBP4	UCHL3	ERH	NFS1		KCTD13	IFIT1	PBXIP1		KRT18	ATP1A1	PSMB4	GAA	EHD1		MAP2K2
H2AZ1	SEMA5A	NQO1	EFNB1	USP39		PCDH12	PRKAR1A	CPNE2		ATP5F1B	EIF2S1	TALDO1	PYGB	ADAM19		COL15A1
EPRS1	DDB1	GLB1	SHROOM1	H3C13		MKS1	LAMP1	CAPNS2		HARS1	IL6	LIPA	ETFA	SH3BGRL3		CTSK
COL4A5	HSPH1	YBX3	COL13A1	PHLDB1		ZNF236	LAMP2	GFM2		PRKCSH	HSPD1	RPS5	ITGA2	PDGFC		LAMB2
EIF2S3	PSMD1	GAP43	H3-7	CCDC47		PSG11	ARSA	AP1B1		AKR1B1	XRCC5	CAPZB	SMPD1	CHRAC1		WASHC4
YARS1	NAP1L4	CTPS1	SIRPB1	LRRC45		ABCA13	ACAN	TWF1		CAPN2	HGF	ARCN1	SDC1	OLA1		TSSK4
EIF3L	SEMA3C	PSMC3	EIF3M	GCC1		TXNDC15	CXCR2	KIF5A		FST	HNRNPL	SARS1	ATP2B1	DPP3		SECTM1
COPG1	LRRC37A2	EIF2S2	COPS6	ST6GAL2		PPARD	CTSS	TRAF2		CTNNA1	VCAM1	RAB7A	PTMS	PSME2		IL1RAP
LRRC59	CROCC2	BTF3	TAOK1	DCBLD2		HGFAC	S100A4	KEAP1		COPB2	U2AF2	COPB1	VDAC1	MAN2B2		CDC23
PGM2	PSMD12	CD9	MOB1B	MYADM		CNTN1	DPP4	PSME4		BSG	MAT2A	RARS1	PCMT1	REXO2		ADAMTS5
HNRNPU	EIF3F	PTPRG	MAMDC2	METRNL		EIF4A2	LAP3	PON2		CAPZA1	STIP1	BCAT1	RPS12	FARP1		FJX1
PRDX4	PSMD14	DDX6	ZDHHC17	FKBP3		SPARCL1	SERPINB1	MAPRE1		ACLY	TIMP3	NAPA	MAP4	CPQ		MYDGF
EIF3A	KPNA3	PSMB5	FAM114A1	CTBS		ITIH4	RNASE4	UAP1		PLTP	FUS	CDC42	PFN2	ALYREF		
CAPRIN1	KPNA4	CMPK1	ALDH16A1	SRSF1		NPTX1	SDC2	IFI16		ADAMTS12	PPP1CC	PSME3	CTNNB1	AP2M1		
ELAVL1	C2CD2L	PPP2R1A	ENAH	PPP3CA		SRSF7	CD86			DSTN	GARS1	RPS4X	GLRX	SCMH1		

Figure S4. Comparative analysis of secretome profiles of perinatal MSCs. A) Principal component analysis (PCA) of the proteome profiles across all placental MSC samples. The PCA score plots showed the separation of the three MSC types (hUCMSCs, hDCMSCs, and hCVMSCs) based on the first two principal components; **B)** The list of uniquely expressed secretory proteins from each MSC group and common proteins shared by two types of MSCs.

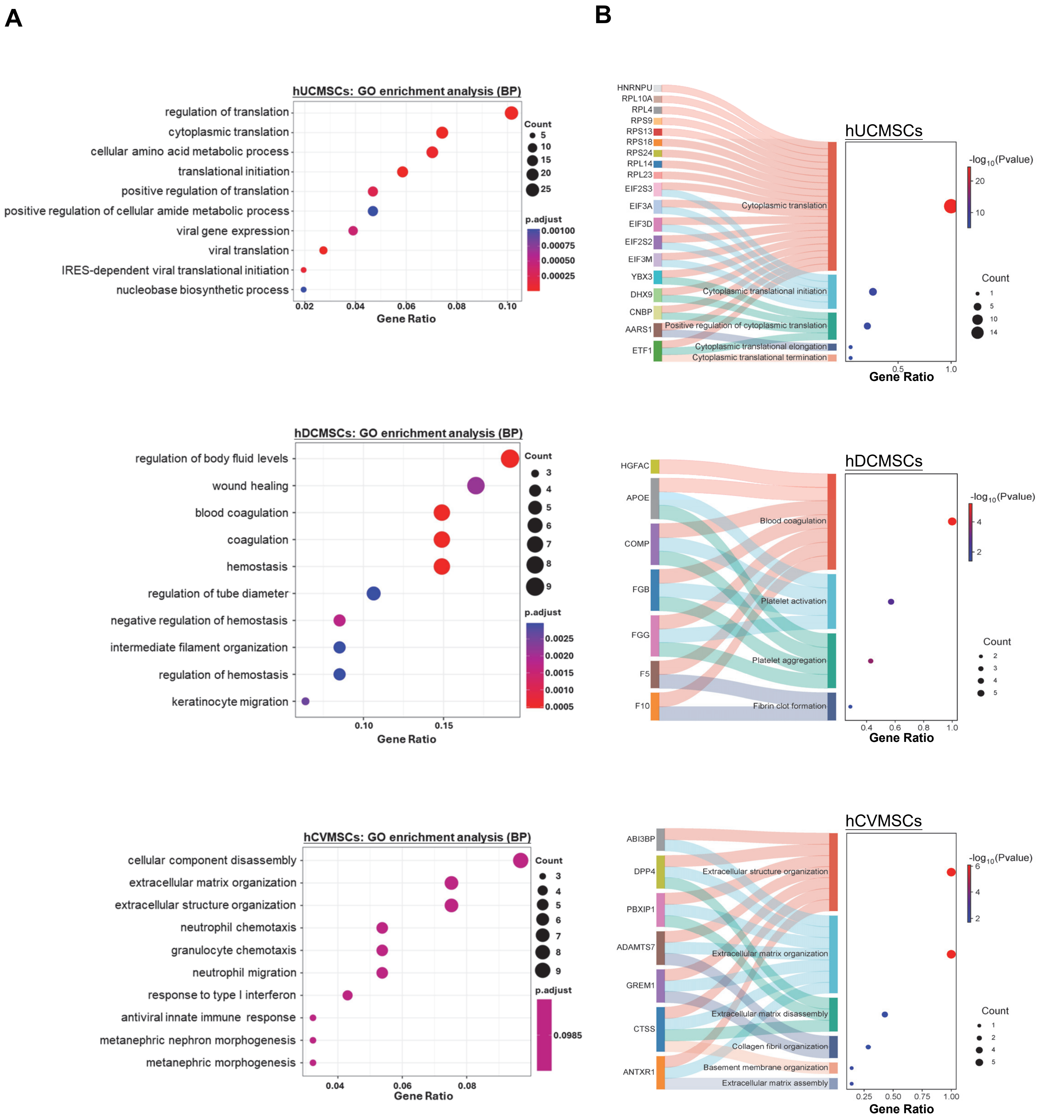
A
hUCMSCs: GO enrichment analysis (BP)
regulation of translation
cytoplasmic translation
cellular amino acid metabolic process
translational initiation
positive regulation of translation
positive regulation of cellular amide metabolic process
viral gene expression
viral translation
IRES-dependent viral translational initiation
nucleobase biosynthetic process
Gene Ratio
Count
p.adjust
hDCMSCs: GO enrichment analysis (BP)
regulation of body fluid levels
wound healing
blood coagulation
coagulation
hemostasis
regulation of tube diameter
negative regulation of hemostasis
intermediate filament organization
regulation of hemostasis
keratinocyte migration
hCVMSCs: GO enrichment analysis (BP)
cellular component disassembly
extracellular matrix organization
extracellular structure organization
neutrophil chemotaxis
granulocyte chemotaxis
neutrophil migration
response to type I interferon
antiviral innate immune response
metanephric nephron morphogenesis
metanephric morphogenesis
B
hUCMSCs
HNRNPU
RPL10A
RPL4
RPS9
RPS13
RPS18
RPS24
RPL14
RPL23
EIF2S3
EIF3A
EIF3D
EIF2S2
EIF3M
YBX3
DHX9
CNBP
AARS1
ETF1
Cytoplasmic translation
Cytoplasmic translational initiation
Positive regulation of cytoplasmic translation
Cytoplasmic translational elongation
Cytoplasmic translational termination
-log10(Pvalue)
Count
Gene Ratio
hDCMSCs
HGFAC
APOE
COMP
FGB
FGG
F5
F10
Blood coagulation
Platelet activation
Platelet aggregation
Fibrin clot formation
hCVMSCs
ABI3BP
DPP4
PBXIP1
ADAMTS7
GREM1
CTSS
ANTXR1
Extracellular structure organization
Extracellular matrix organization
Extracellular matrix disassembly
Collagen fibril organization
Basement membrane organization
Extracellular matrix assembly

Figure S5. Analysis of secretome profiles. A) GO analysis of biological processes for the individually expressed proteins in hUCMSCs, hCVMSCs and hDCMSCs; **B)** Sankey dot plot of regulation of translation (hUCMSCs), blood coagulation (hDMSCs) and extracellular matrix organization (hCVMSCs).

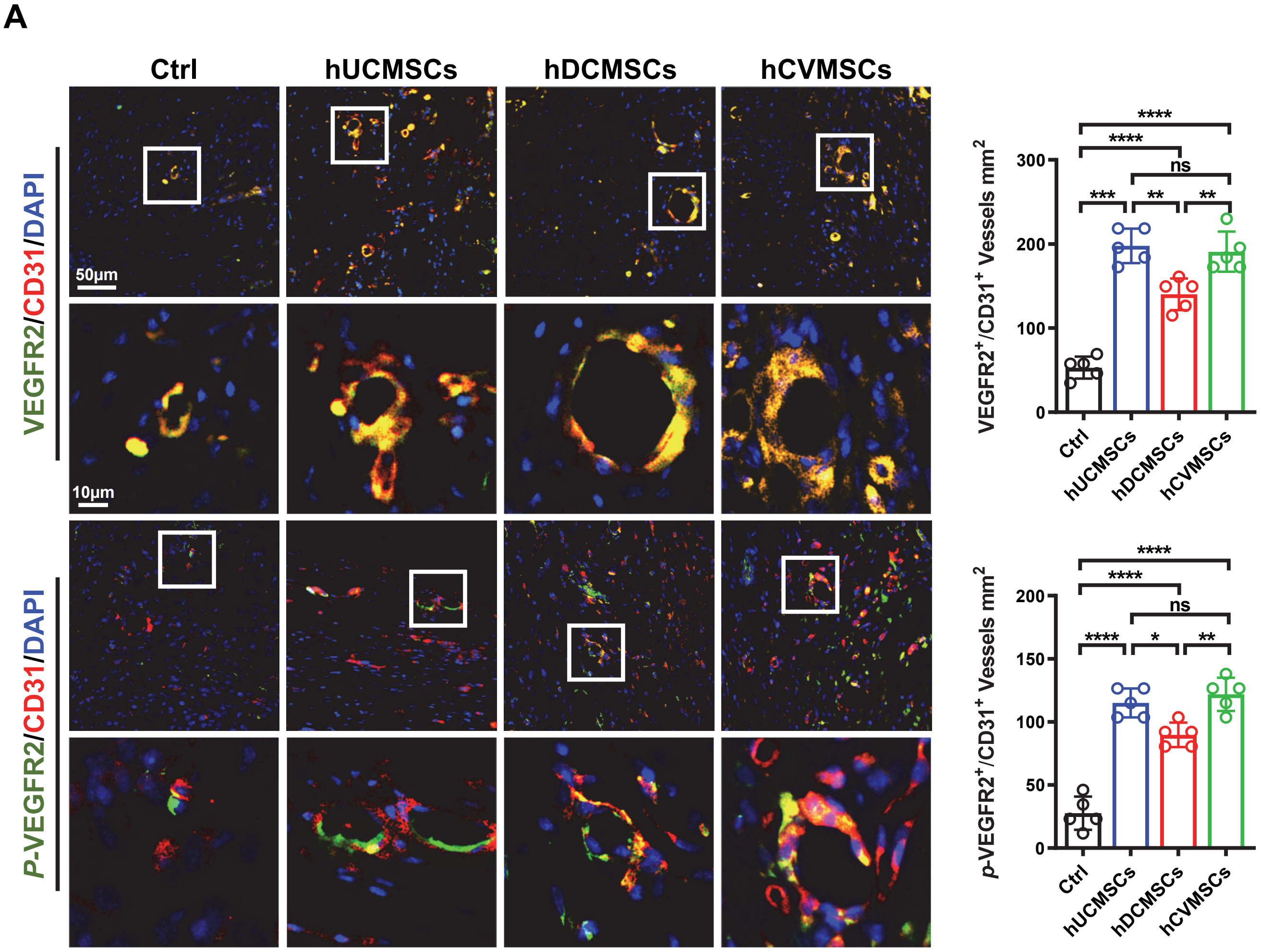
A
Ctrl
hUCMSCs
hDCMSCs
hCVMSCs
VEGFR2/CD31/DAPI
50μm
10μm
p-VEGFR2/CD31/DAPI
VEGFR2+/CD31+ Vessels mm2
0
100
200
300
****
****
ns
***
**
**
Ctrl
hUCMSCs
hDCMSCs
hCVMSCs
p-VEGFR2+/CD31+ Vessels mm2
0
50
100
150
200
****
****
ns
****
*
**
Ctrl
hUCMSCs
hDCMSCs
hCVMSCs

Figure S6. Human perinatal MSCs promote VEGFR signaling in endothelial cells. Representative immunofluorescence (IF) images of VEGFR2, p-VEGFR2 and CD31 staining in wounds on day 10 for each group. Quantification of double positive cells per unit area is shown on the right. All data are presented as mean ± SD, n = 5 for all groups. Scale bar = 50 μm and 10 μm. *, **, *** and **** represent $p < 0.05$, 0.01,0.001, and 0.0001, respectively, by Tukey's *post-hoc* test when statistical significance by One-way ANOVA ($p<0.05$) is obtained.

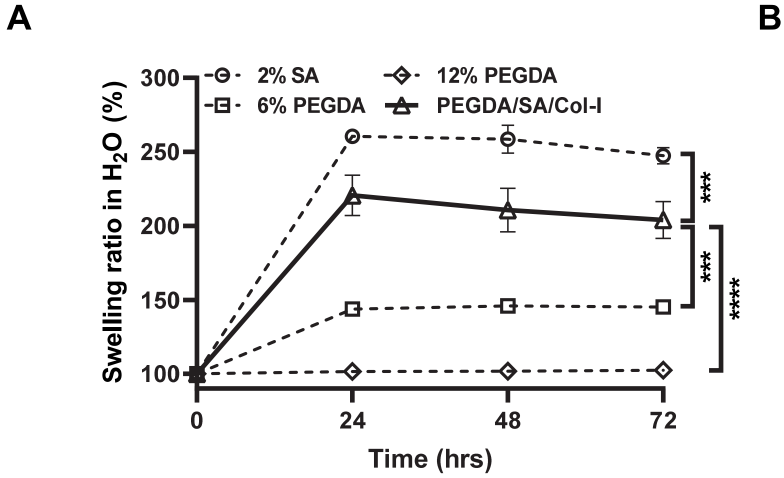
A
2% SA
6% PEGDA
12% PEGDA
PEGDA/SA/Col-I
Swelling ratio in H2O (%)
300
250
200
150
100
0
24
48
72
Time (hrs)
***
***
****
B
2% SA
6% PEGDA
12% PEGDA
PEGDA/SA/Col-I
Swelling ratio in PBS (%)
300
250
200
150
100
0
24
48
72
Time (hrs)
****
***

Figure S7. Assessment of swelling properties of various hydrogels in H_2O and PBS. The swelling ratio of different hydrogels were determined for 72 hours in H_2O and PBS. Experiments were repeated at least three times. All data are presented as mean ± SD. ***, and **** represent $p < 0.001$ and 0.0001, respectively, by Tukey's *post-hoc* test when statistical significance by One-way ANOVA ($p<0.05$) is obtained.

HaCaT

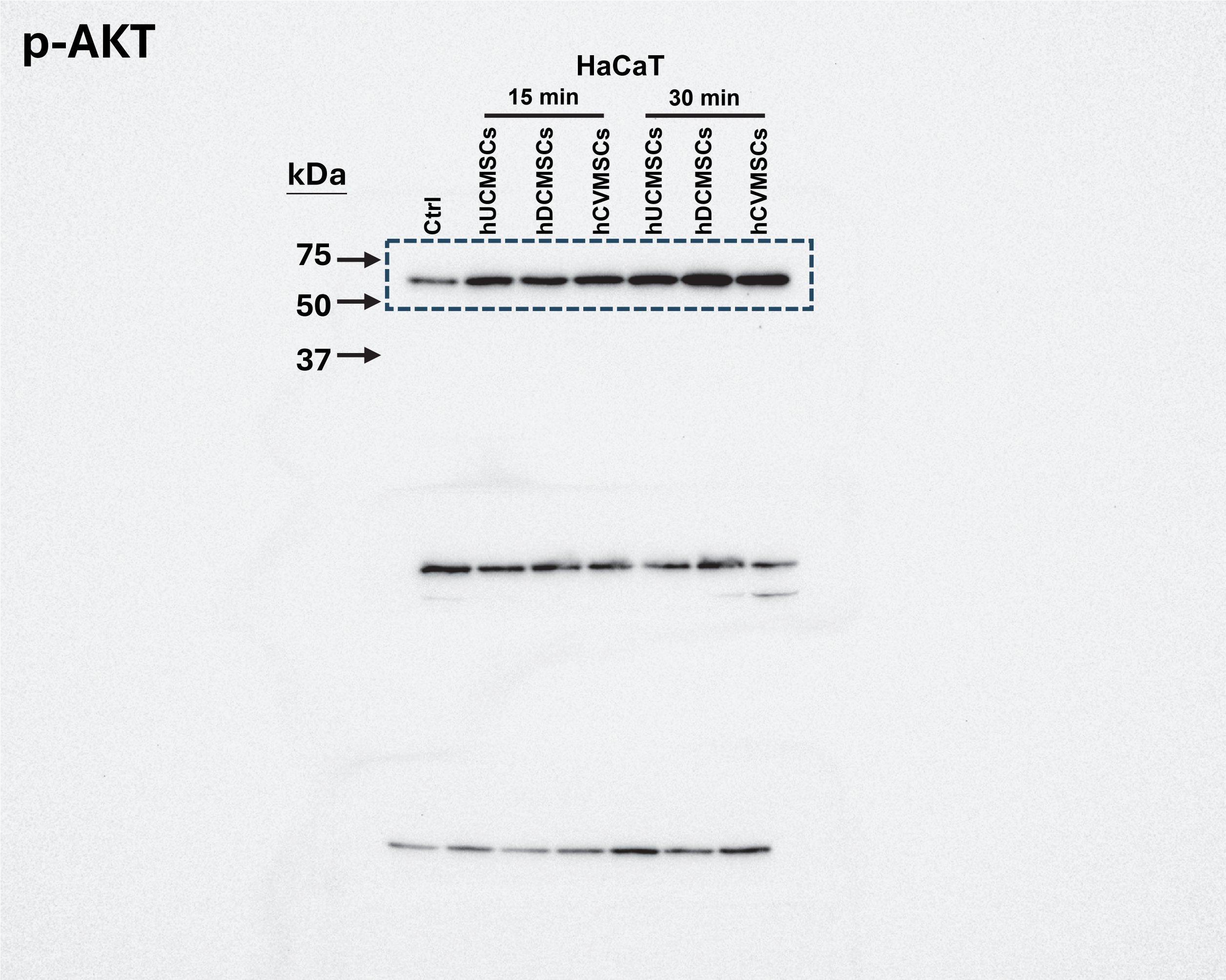
p-AKT
HaCaT
15 min
30 min
kDa
Ctrl
hUCMSCs
hDCMSCs
hCVMSCs
hUCMSCs
hDCMSCs
hCVMSCs
75
50
37

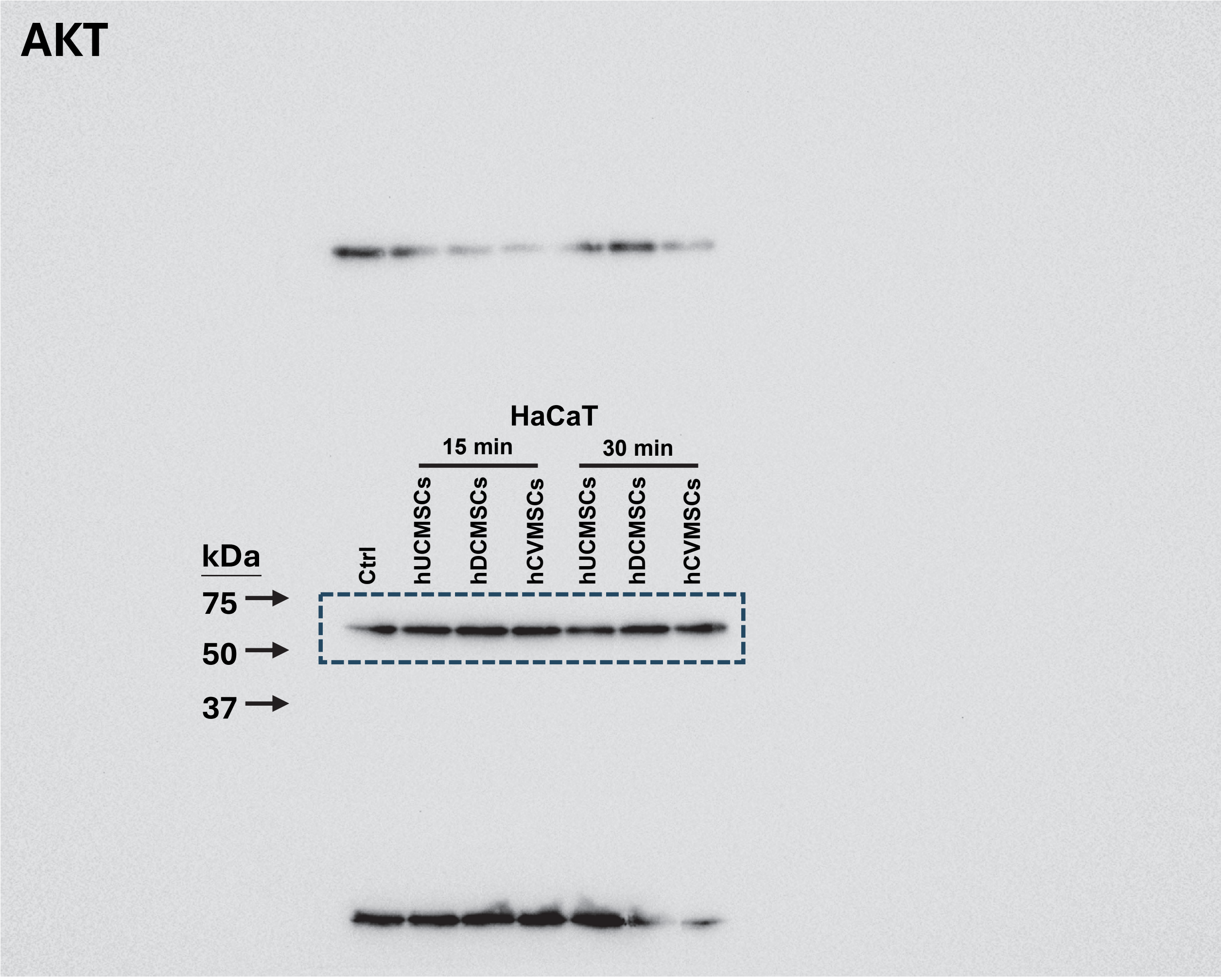
AKT
HaCaT
15 min
30 min
kDa
Ctrl
hUCMSCs
hDCMSCs
hCVMSCs
hUCMSCs
hDCMSCs
hCVMSCs
75
50
37

HaCaT

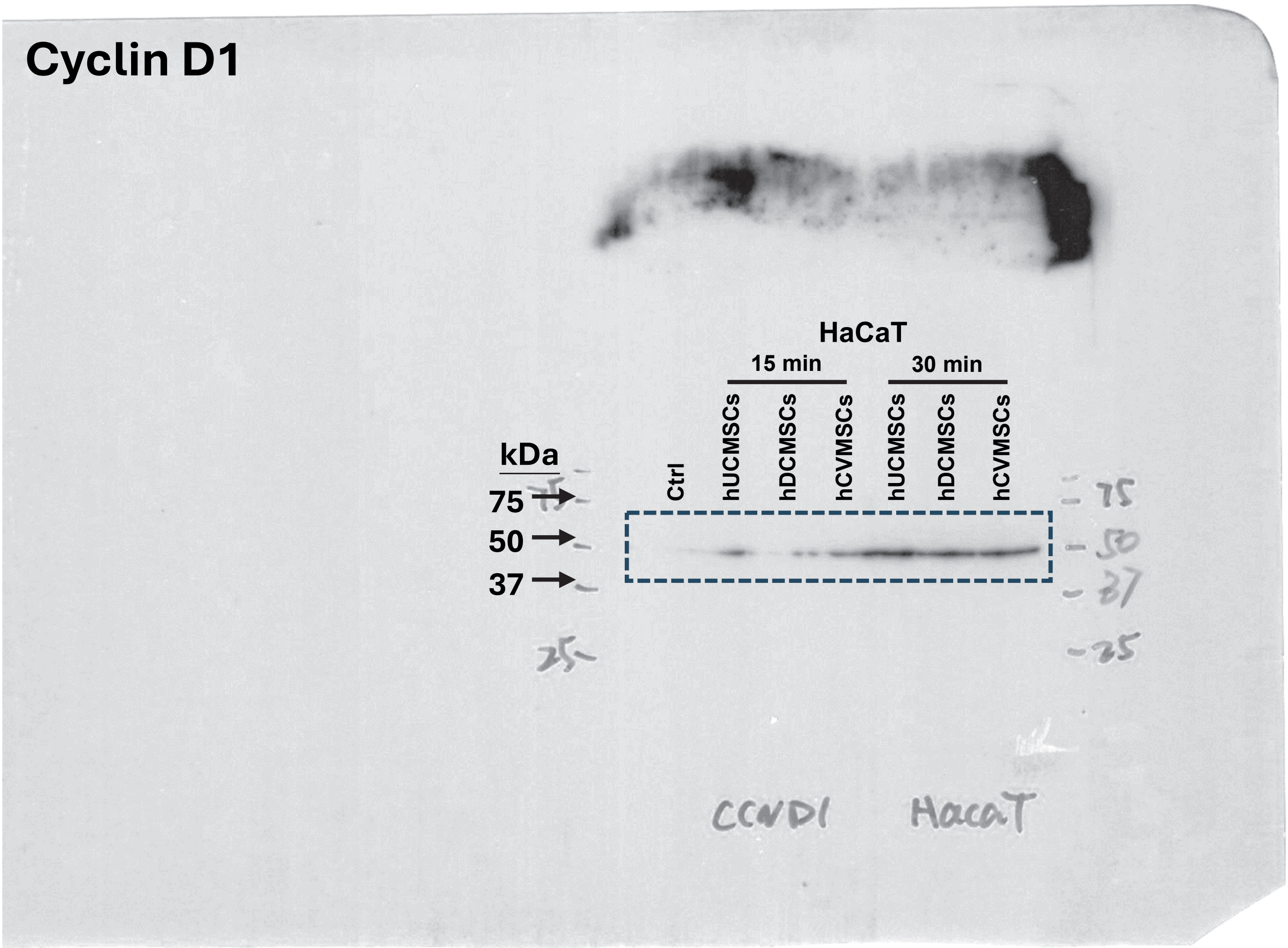
Cyclin D1
HaCaT
15 min
30 min
kDa
75
50
37
Ctrl
hUCMSCs
hDCMSCs
hCVMSCs
hUCMSCs
hDCMSCs
hCVMSCs
75
50
37
25
CCND1
HacaT

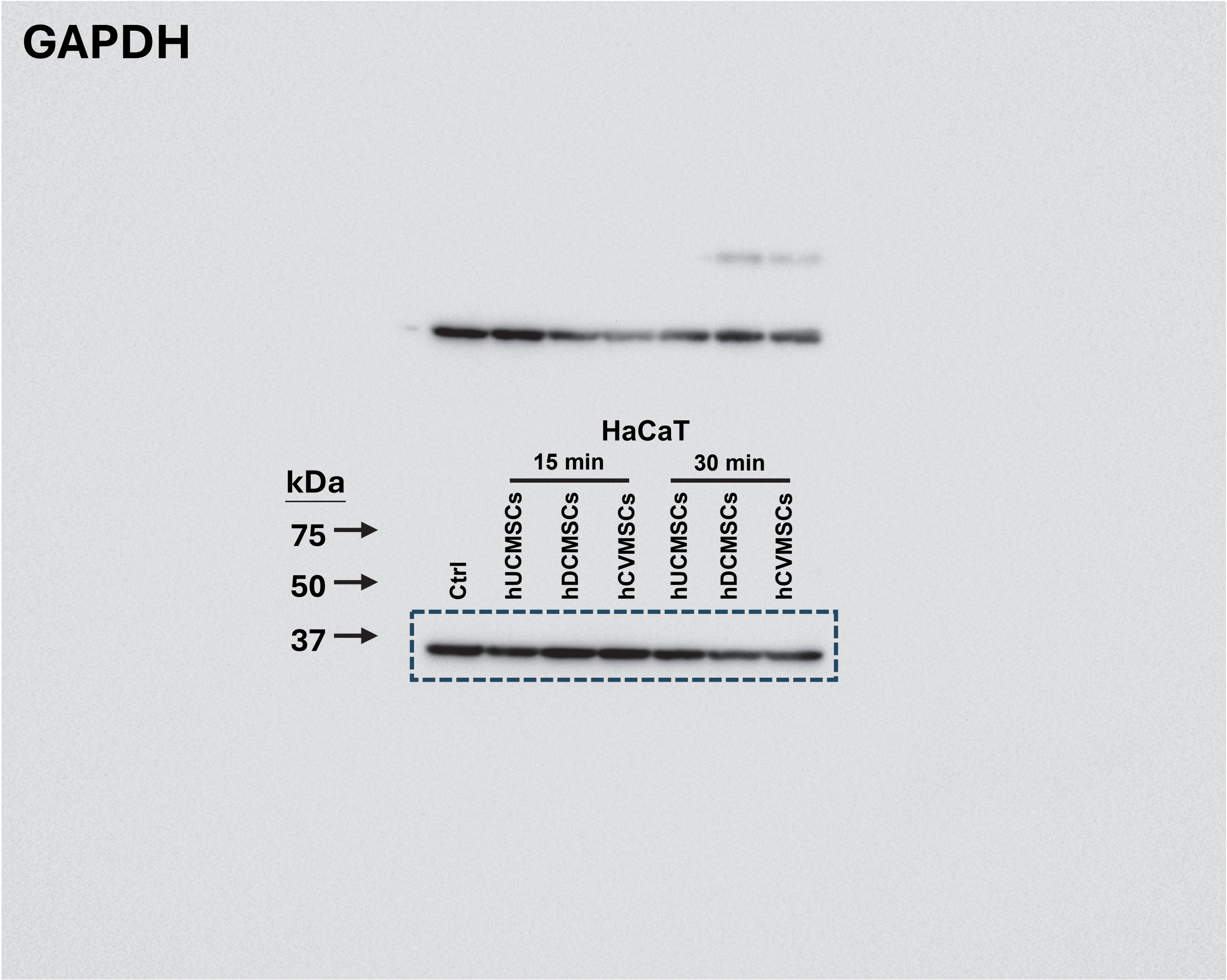
GAPDH
HaCaT
15 min
30 min
kDa
75
50
37
Ctrl
hUCMSCs
hDCMSCs
hCVMSCs
hUCMSCs
hDCMSCs
hCVMSCs

HUVEC

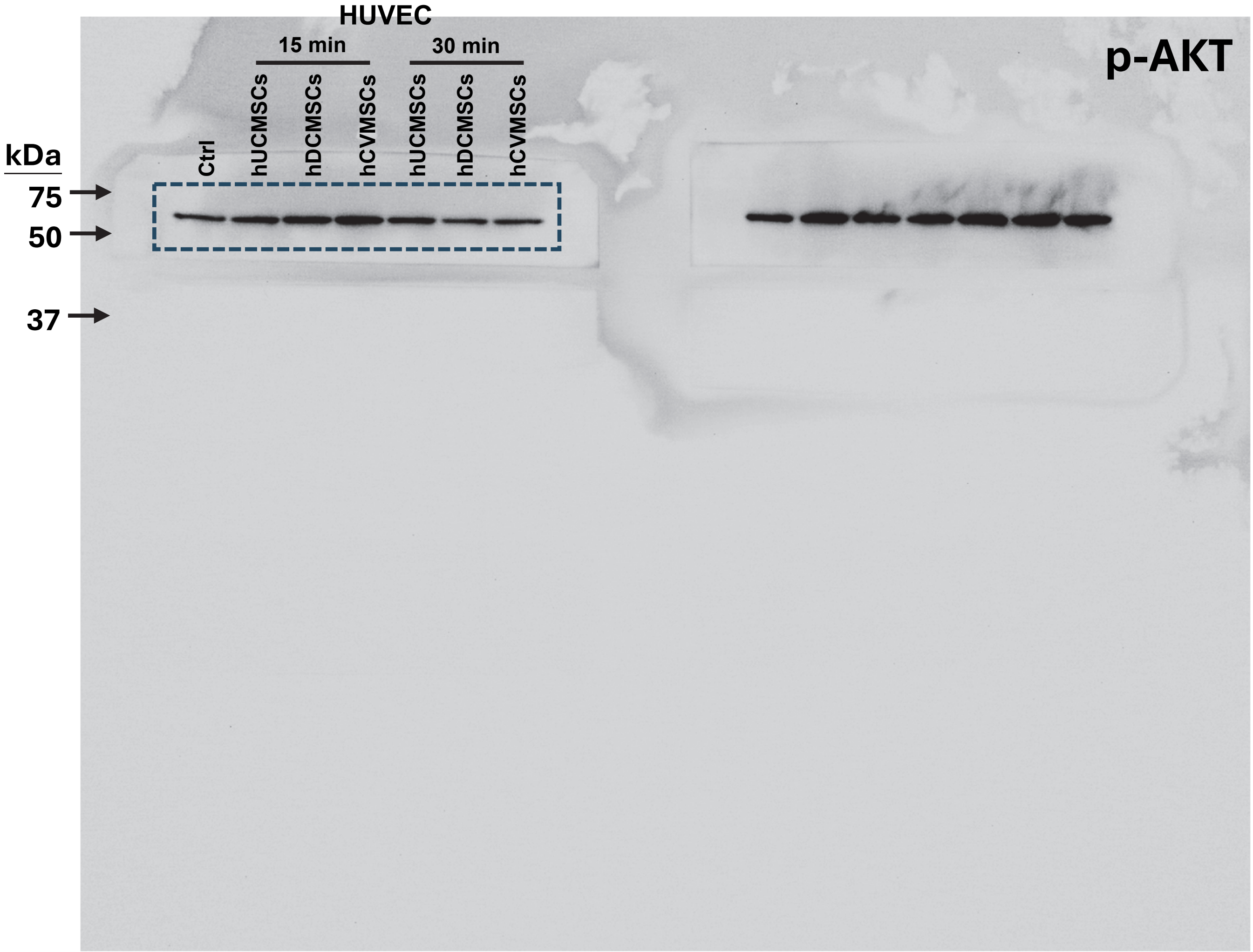
HUVEC
15 min
30 min
p-AKT
kDa
75
50
37
Ctrl
hUCMSCs
hDCMSCs
hCVMSCs
hUCMSCs
hDCMSCs
hCVMSCs

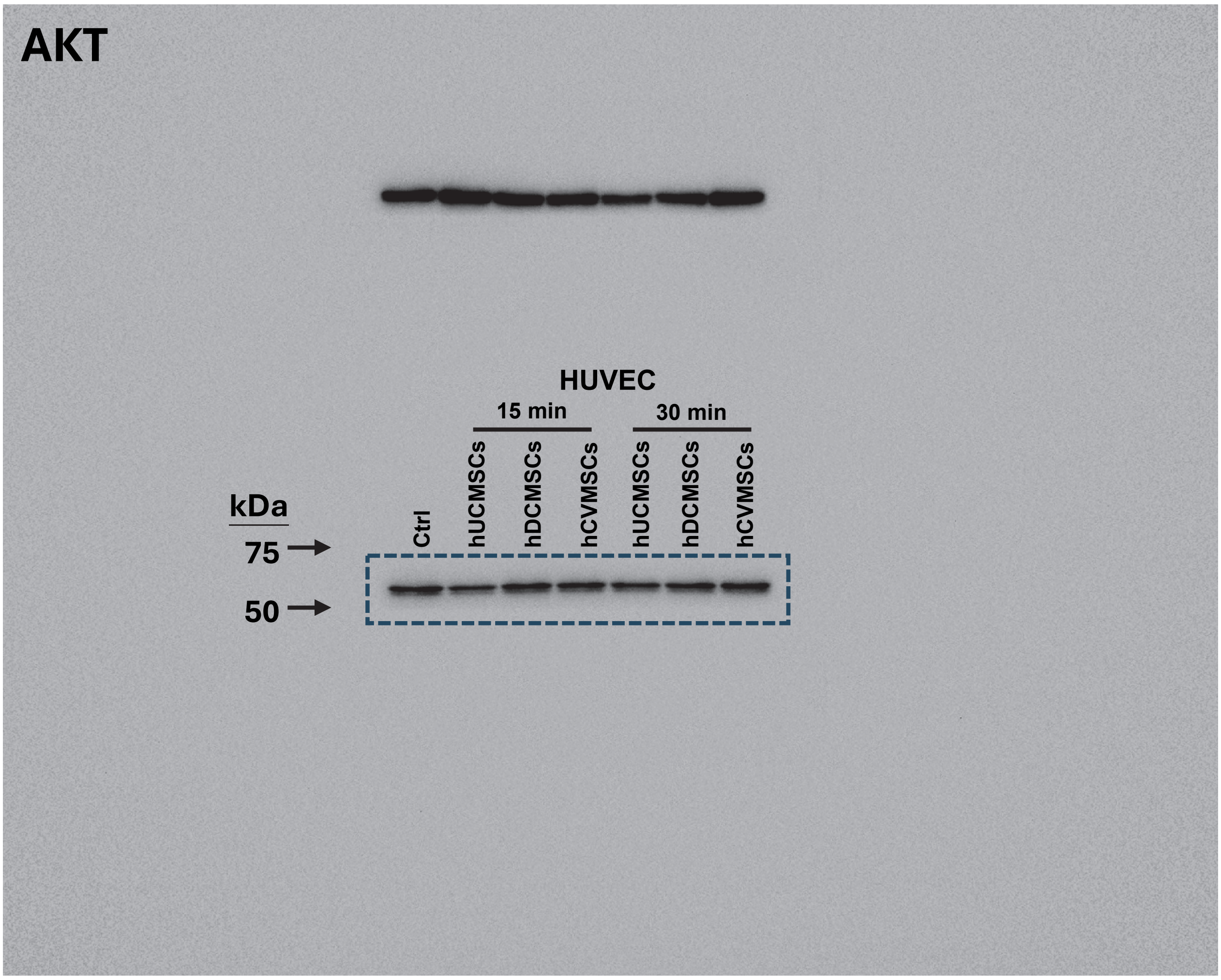
AKT
HUVEC
15 min
30 min
kDa
75
50
Ctrl
hUCMSCs
hDCMSCs
hCVMSCs
hUCMSCs
hDCMSCs
hCVMSCs

HUVEC

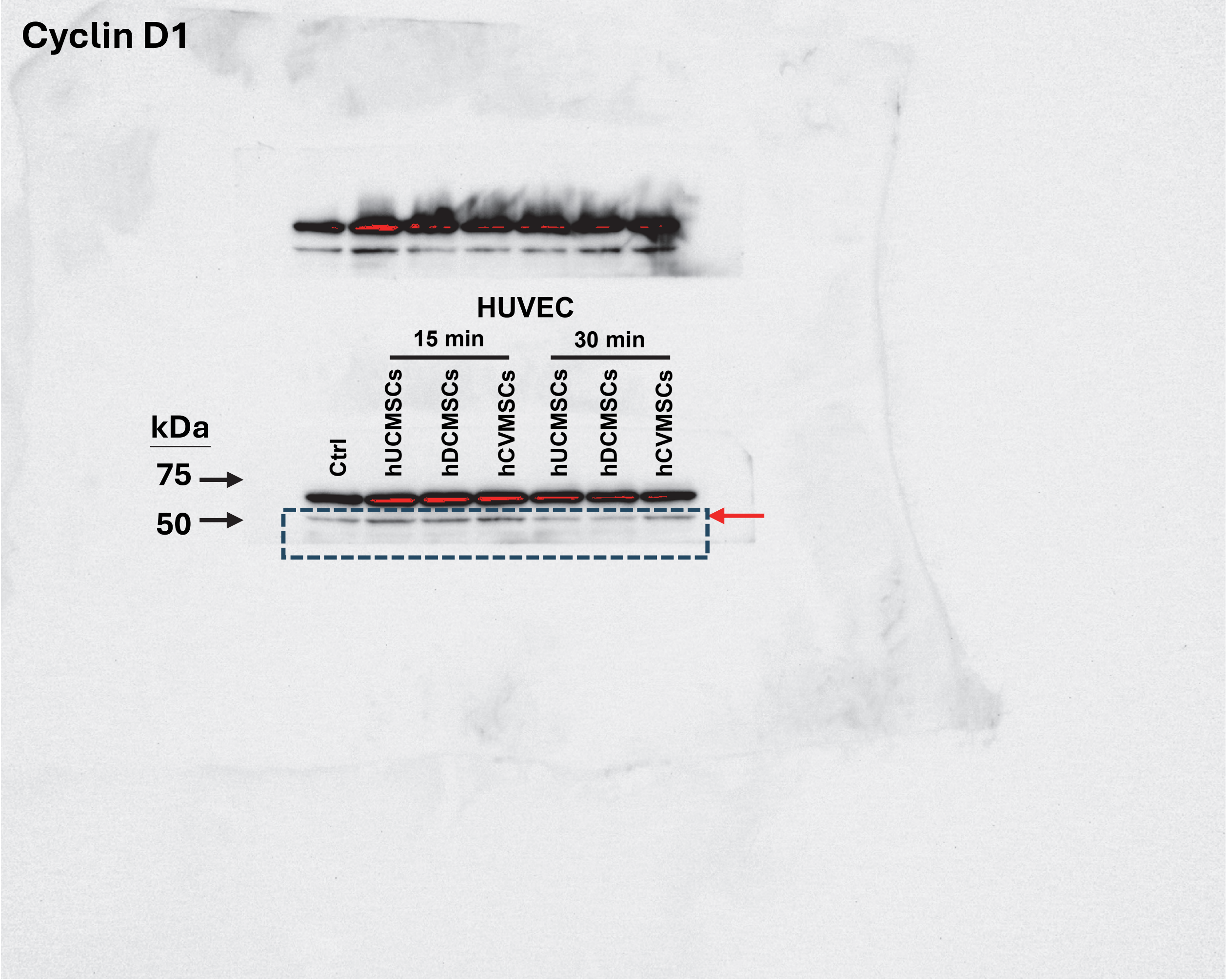

Cyclin D1
HUVEC
15 min
30 min
kDa
75
50
Ctrl
hUCMSCs
hDCMSCs
hCVMSCs
hUCMSCs
hDCMSCs
hCVMSCs

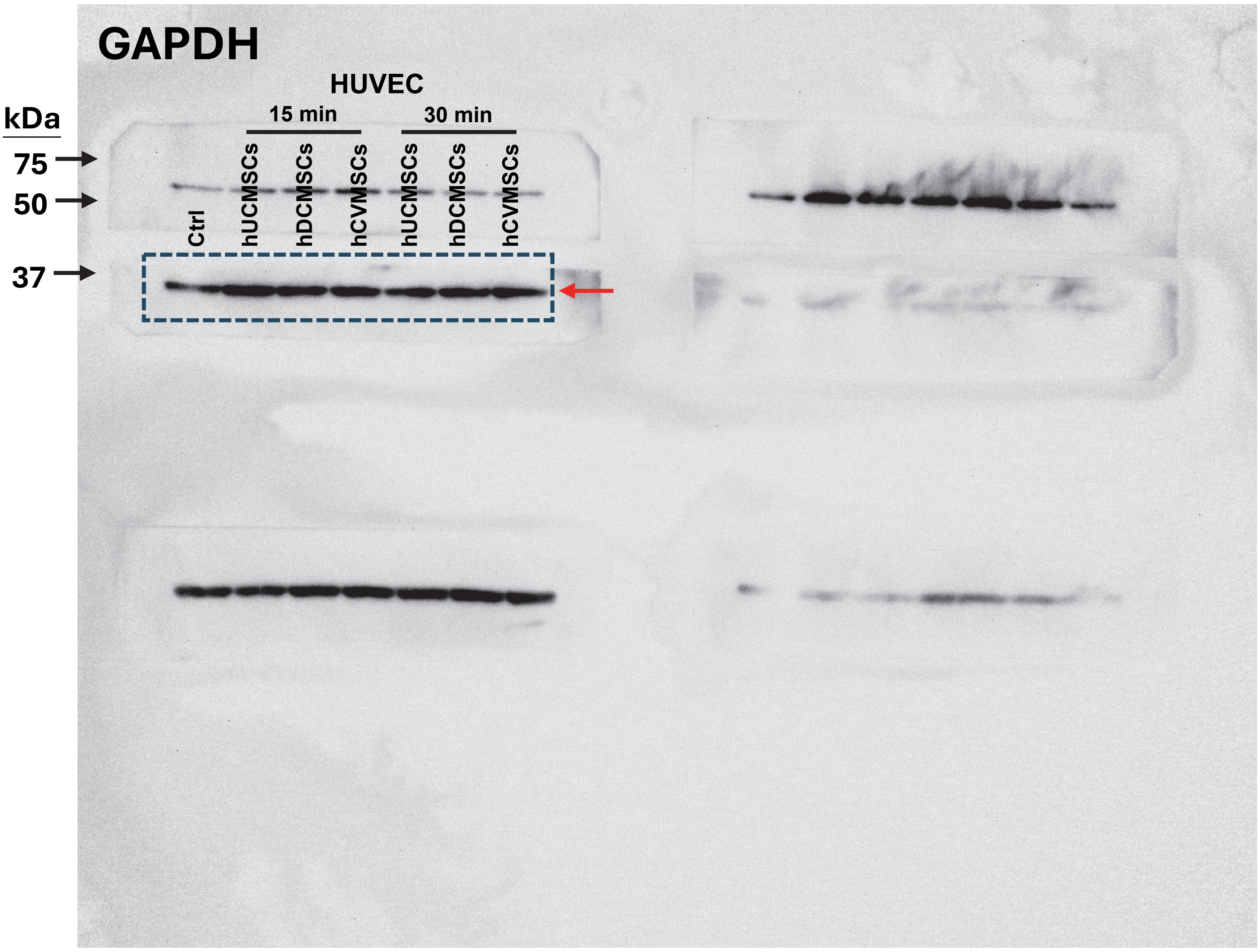

GAPDH
HUVEC
15 min
30 min
kDa
75
50
37
Ctrl
hUCMSCs
hDCMSCs
hCVMSCs
hUCMSCs
hDCMSCs
hCVMSCs

Figure S8. Full-length western blots for Figure 5.
